# Papain-like and legumain-like proteases in rice: genome-wide identification, comprehensive gene feature characterization and expression analysis

**DOI:** 10.1186/s12870-018-1298-1

**Published:** 2018-05-15

**Authors:** Wei Wang, Xue-mei Zhou, Han-xian Xiong, Wan-ying Mao, Peng Zhao, Meng-xiang Sun

**Affiliations:** State Key Laboratory of Hybrid Rice, College of Life Sciences, Wuhan University, Wuhan, 430072 China

**Keywords:** Papain-like protease, Legumain-like protease, Plant hormones, Stress, Rice

## Abstract

**Background:**

Papain-like and legumain-like proteases are proteolytic enzymes which play key roles in plant development, senescence and defense. The activities of proteases in both families could be inhibited by a group of small proteins called cystatin. Cystatin family genes have been well characterized both in tobacco and rice, suggesting their potential roles in seed development. However, their potential targets, papain-like and legumain-like proteases, have not been well characterized in plants, especially in rice, a model plant for cereal biology.

**Results:**

Here, 33 papain-like and 5 legumain-like proteases have been identified in rice genome, respectively. Gene structure, distribution in rice chromosome, and evolutionary relationship to their counterparts in other plants have been well characterized. Comprehensive expression profile analysis revealed that two family genes display divergent expression pattern, which are regulated temporally and spatially during the process of seed development and germination. Our experiments also revealed that the expression of most genes in these two families is sensitively responsive to plant hormones and different abiotic stresses.

**Conclusions:**

Genome-wide identification and comprehensive gene expression pattern analysis of papain-like and legumain-like proteases in rice suggests their multiple and cooperative roles in seed development and response to environmental variations, which provides several useful cues for further in-depth study.

**Electronic supplementary material:**

The online version of this article (10.1186/s12870-018-1298-1) contains supplementary material, which is available to authorized users.

## Background

Plant genomes encode hundreds of proteases, which belong to dozens of unrelated families and have been divided into different families and clans on the basis of evolutionary relationships. Among these proteases, papain-like cysteine proteases (PLCPs) in peptidase C1A family and legumain-like cysteine proteases (LLCPs) in peptidase C13 family [1] are known as two specific types of cysteine proteases, whose activities could both be inhibited by a group of small proteins called cystatin [2, 3].

PLCPs contain two domains: an α-helix and β-sheet which delimit a cleft at the surface acting as the substrate-binding groove [4] and a catalytic triad Cys-His-Asn which is highly conserved among different kingdoms. PLCPs are encoded as inactive precursors, which comprise an N-terminal signal peptide, a prodomain and the mature protein. By limited intra- or inter-molecular proteolysis, after cleaving off an inhibitory propeptide in an acidic environment [5], PLCPs become active [6] and function in various physiological processes such as seed germination [7, 8], male organ development [9–12], senescence [13], defense against pathogens [14, 15], and response to insect attack or abiotic stress [16, 17].

LLCPs are a group of Asn-specific proteinases, which were primarily located in the vacuole and responsible for maturation of storage proteins in seeds [18–20]. Considering their intracellular localizations and function, LLCPs are also named ‘vacuolar processing enzymes’ (VPEs) [21]. Similar to PLCPs, VPEs are usually synthesized as inactive precursors composed of a short N- and a much longer C-terminal propeptide flanking the mature enzyme [21, 22]. VPE is usually self-catalytically converted into the mature form at acidic condition by sequential removal of the C-terminal propetide and N-terminal propetide, which is an essential step for enzyme activation [18, 23]. VPEs have been shown to participate in protein processing in several physiological processes, which are responsible for maturation and/or activation of various vacuolar proteins [20, 24]. In addition, several VPEs have also been shown to function in regulating programmed cell death (PCD) both in developmental process and defense responses through their caspase-like activities [25–28].

Rice (*Oryza sativa* L.) is one of most widely grown crop in the world, which provides main food source for people in Southeast Asia, and has been considered as model species for many basic and applied researches. Great efforts have been made to improve rice yield and resistance to different biotic and abiotic stresses [29–32]. As described above, PLCPs and LLCPs were reported to be involved in seed development and plant defense against different stresses. However, few of them have been well characterized, especially in rice [11, 12, 33]. Thus, genome wide identification and expression analysis of PLCPs and LLCPs is helpful to explore their potential roles in rice seed development, and improve rice yield and resistance to various stresses. Here, 33 PLCPs and 5 LLCPs have been identified and characterized, providing valuable clues to gain insight into their specific physiological roles in the further study.

## Results

### Identification and cloning of *OsCPs* and *OsVPE*s in rice genome

To identify genes encoding PLCPs and LLCPs in rice, tBLASTP program in National Center for Biotechnology Information (NCBI) database was performed using 30 protein sequences of PLCPs in *Arabidopsis thaliana* identified by Beers et al. [34] and 4 protein sequences of LLCPs in *A. thaliana* [27], respectively. After removing redundant sequences, candidates with intact open reading frame covering peptidase C1A and peptidase C13 domain were considered as true *OsCP* and *OsVPE* in rice. These predicted *OsCPs* and *OsVPE*s were further confirmed by PCR using cDNA as templates. Finally, 33 genes (Designated as *OsCP1*-*OsCP33*) encoding PLCP and 5 genes (Designated as *OsVPE1*-*OsVPE5*) encoding LLCP were identified in rice genome, respectively. The information for each gene in rice was listed in Table 1.

**Table 1:** Detailed information of *OsCPs* and *OsVPEs*

Gene	RAP-DB Locus ID	TIGR/MSU Locus ID	Signal peptide	No of amino acid	Mol. Weight (kDa)	PI	Chromosome Location
*OsCP1*	Os04g0670200	LOC_Os04g57440	21	466	49,798.7	5.02	Chr4 34,176,732–34,172,494
*OsCP2*	Os04g0670500	LOC_Os04g57490	31	490	52,642.3	7.61	Chr4 34,205,994–34,207,906
*OsCP3*	Os11g0255300	LOC_Os11g14900	24	378	40,933.2	5.79	Chr11 8,382,036–8,379,342
*OsCP4*	Os05g0108600	LOC_Os05g01810	22	358	39,036.6	4.96	Chr5 486,053–488,406
*OsCP5*	Os01g0971400	LOC_Os01g73980	27	365	39,927.8	5.06	Chr1 42,855,657–42,857,466
*OsCP6*	Os01g0907600	LOC_Os01g67980	28	371	40,719.5	6.64	Chr1 39,501,330–39,502,978
*OsCP7*	Os02g0715000	LOC_Os02g48450	30	366	40,599.9	8.00	Chr2 29,667,743–29,669,662
*OsCP8*	Os08g0556900	LOC_Os08g44270	30	385	41,871.8	6.85	Chr8 27,875,142–27,876,599
*OsCP9*	Os09g0497500	LOC_Os09g32230	24	349	37,379.2	4.76	Chr9 19,240,737–19,239,243
*OsCP10*	Os05g0508300	LOC_Os05g43230	25	450	47,620.8	5.97	Chr5 25,153,902–25,157,655
*OsCP11*	Os04g0203500	LOC_Os04g12660	23	301	33,117.8	7.78	Chr4 7,008,959–7,010,088
*OsCP12*	Os09g0381400	LOC_Os09g21370	26	362	38,639.5	5.19	Chr9 12,899,550–12,897,272
*OsCP13*	Os06g0582600	LOC_Os06g38450	29	357	38,509.2	7.11	Chr6 22,779,409–22,781,109
*OsCP14*	Os01g0347500	LOC_Os01g24550	26	361	38,988.9	6.38	Chr1 13,839,339–13,837,906
*OsCP15*	Os01g0613500	LOC_Os01g42780	30	360	37,513.6	7.52	Chr1 24,345,173–24,343,518
*OsCP16*	Os01g0330200	LOC_Os01g22670	22	357	38,565.5	6.41	Chr1 12,747,081–12,745,130
*OsCP17*	Os01g0217300	LOC_Os01g11840	28	366	39,292.3	5.75	Chr1 6,400,890–6,402,066
*OsCP18*	Os09g0442300	LOC_Os09g27030	23	362	39,114.0	7.16	Chr9 16,439,141–16,443,631
*OsCP19*	Os01g0330300	LOC_Os01g22680	26	367	39,474.7	6.84	Chr1 12,751,899–12,753,162
*OsCP20*	Os02g0469600	LOC_Os02g27030	19	373	40,591.6	6.44	Chr2 15,891,528–15,895,297
*OsCP21*	Os04g0208200	LOC_Os04g13140	26	349	36,867.3	5.10	Chr4 7,250,358–7,251,846
*OsCP22*	Os04g0107700	LOC_Os04g01710	31	383	41,996.4	5.57	Chr4 472,891–471,100
*OsCP23*	Os12g0273800	LOC_Os12g17540	24	350	37,170.8	4.79	Chr12 10,053,411–10,055,200
*OsCP24*	Os09g0562700	LOC_Os09g38920	32	382	40,882.8	6.07	Chr9 22,353,654–22,351,648
*OsCP25*	Os01g0347600	LOC_Os01g24560	29	343	37,322.2	6.90	Chr1 13,842,272–13,843,749
*OsCP26*	Os09g0564600	LOC_Os09g39110	26	374	41,375.9	5.49	Chr9 22,453,266–22,450,380
*OsCP27*	Os01g0613800	LOC_Os01g42790	22	359	38,250.9	7.71	Chr1 24,353,345–24,351,302
*OsCP28*	Os07g0480900	LOC_Os07g29760	22	376	39,868.1	6.39	Chr7 17,496,502–17,498,225
*OsCP29*	Os09g0565100	LOC_Os09g39170	24	319	34,267.0	5.99	Chr9 22,494,320–22,491,705
*OsCP30*	Os04g0311400	LOC_Os04g24600	24	381	41,555.7	6.51	Chr4 14,129,614–14,132,611
*OsCP31*	Os09g0564000	LOC_Os09g39060	19	283	30,472.5	4.93	Chr9 22,422,558–22,420,898
*OsCP32*	Os09g0564200	LOC_Os09g39070	25	369	39,515.8	4.98	Chr9 22,430,508–22,427,465
*OsCP33*	Os05g0310500	LOC_Os05g24550	22	434	48,152.4	7.09	Chr5 14,161,386–14,167,285
*OsVPE1*	Os01g0559600	LOC_Os01g37910	29	501	54,964.9	5.80	Chr1 21,230,393–21,234,929
*OsVPE2*	Os04g0537900	LOC_Os04g45470	23	497	54,885.8	5.14	Chr4 26,908,679–26,903,866
*OsVPE3*	Os02g0644000	LOC_Os02g43010	22	496	54,847.9	5.48	Chr2 25,895,244–25,890,441
*OsVPE4*	Os05g0593900	LOC_Os05g51570	19	474	51,845.3	5.72	Chr5 29,580,698–29,579,044
*OsVPE5*	Os02g0219400	LOC_Os02g12740	35	404	45,123.2	5.70	Chr2 6,672,950–6,668,822

### Gene structure analysis of *OsCPs* and *OsVPE*s

**Fig. 1:**
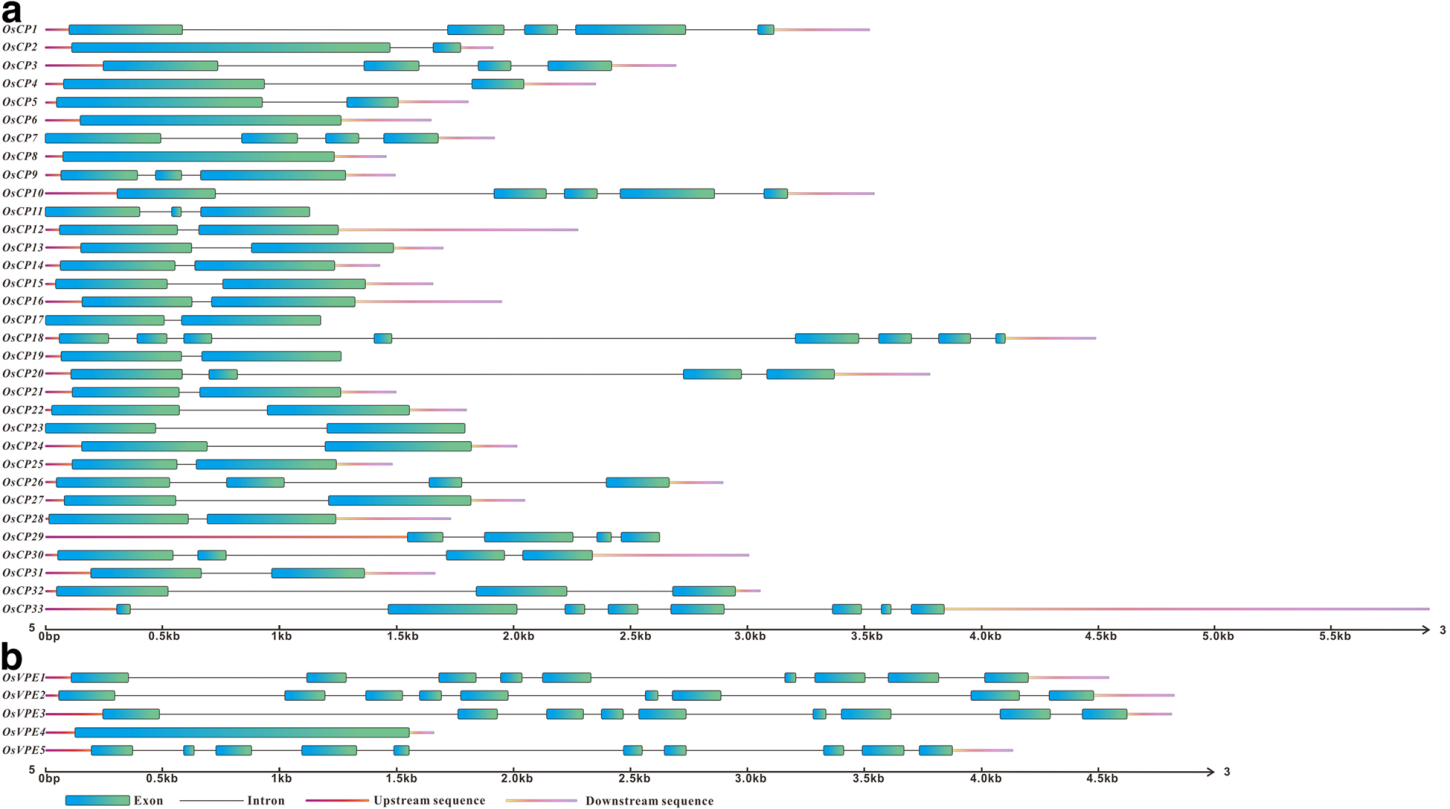
Genomic structure of *OsCPs* and *OsVPEs* in rice. **a** and **b** correspond to *OsCPs* and *OsVPEs* respectivily

Intron-exon structure of *OsCPs* and *OsVPEs* were determined by comparison of the cDNA sequences with their corresponding genomic sequences. The results revealed that the genes encoding PLCPs could be divided into three groups according to the number of intron (Fig. 1a). The first group consists of *OsCPs* without any intron. Two *OsCPs* (O*sCP6* and *OsCP8*) belong to this group. The second group is single-intron gene, and above half of *OsCPs* (*OsCP2*, *OsCP4*, *OsCP5*, *OsCP12*, *OsCP13*, *OsCP14*, *OsCP15*, *OsCP16*, *OsCP17*, *OsCP19*, *OsCP21*, *OsCP22*, *OsCP23*, *OsCP24*, *OsCP25*, *OsCP27*, *OsCP28* and *OsCP31*) fall into the second group. The third group is multiple-intron *OsCPs*, and the remaining *OsCPs* (*OsCP1*, *OsCP3*, *OsCP7*, *OsCP9*, *OsCP10*, *OsCP11*, *OsCP18*, *OsCP20*, *OsCP26*, *OsCP29*, *OsCP30*, *OsCP32* and *OsCP33*) belong to the third group. The number of introns in the third group is divergent, ranging from two to seven (Fig. 1a). As for *OsVPE*s, most *OsVPEs* harbored multiple introns with one exception (*OsVPE4*) (Fig. 1b). However, the length of coding sequences of *OsCPs* and *OsVPEs* seems conserved, with 852 to 1473 nucleotides for *OsCPs* and 1215 to 1506 nucleotides for *OsVPEs*, indicating that divergent number and length of intron determine the gene size of two families in genome.

### Chromosomal localization and gene duplication analysis

**Fig. 2:**
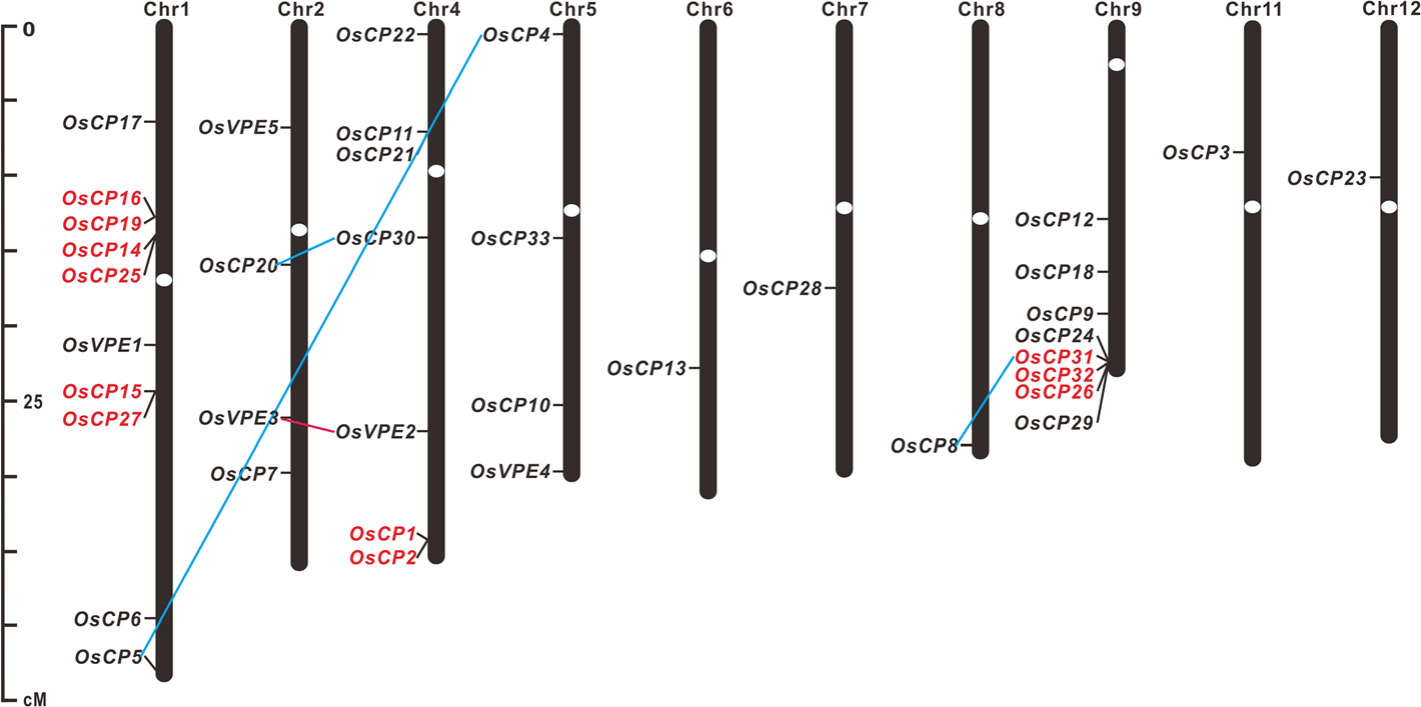
Localization of *OsCPs* and *OsVPEs* on rice chromosomes. Chromosome number was indicated at the top of each chromosome. The size of chromosome was labeled on the left of the figure. Tandem duplicated genes were outlined with red color, and segmental duplicated gene pairs were linked with blue and red lines

Physical locations of these two families in the rice chromosomes were determined according to their genome sequences. 33 *OsCPs* were mapped to 10 rice chromosomes with an uneven distribution pattern (Fig. 2). Majority of *OsCPs* were assigned to chromosome 1, 4 or 9, with 6–9 *OsCPs* in each chromosome. The distribution of remaining *OsCPs* was scattered, with one to three genes in each chromosome. As for *OsVPEs*, they were assigned to four chromosomes (Fig. 2). Chromosome 2 contains two genes (*OsVPE3* and *OsVPE5*), and chromosome 1, 4, 5 harbor one *VPE* respectively.

Furthermore, gene duplication events of *OsCP* and *OsVPE* families during long evolutionary history were also analyzed. Gene pairs separated at most by five intervening genes were considered as tandem duplicates [35]. There are five pairs located in tandem repeats (*OsCP1*/*OsCP2*, *OsCP14*/*OsCP25*, *OsCP16*/*OsCP19*, *OsCP15*/*OsCP27* and *OsCP26*/*OsCP31*/*OsCP32*) in *OsCP* family (Fig. 2). However, no gene tandem duplication events was found in *OsVPE* family. At same time, three pairs (*OsCP4*/*OsCP5*, *OsCP8*/*OsCP31* and *OsCP20*/*OsCP30*) from *OsCP* family and one pair (*OsVPE2*/*OsVPE3*) from *OsVPE* family were found to be present on the duplicated chromosomal segments, suggesting that *OsCPs* in rice expanded through both segmental and tandem duplications, but *OsVPEs* only through segmental duplications.

Ka/Ks ratio, a tool to measure gene divergence, was also calculated in the present study. Ka/Ks < 1 means negative selection; Ka/Ks = 1 means neutral selection, and Ka/Ks > 1 means positive selection [36]. Duplicated pairs (*OsCP1/OsCP2*, *OsCP14/OsCP25*, *OsCP16/OsCP19*, *OsCP15/OsCP27* and *OsCP26/OsCP31*) were suggested to belong to positive selection as indicated from Ka/Ks value, and Ka/Ks ratios of the remaining pairs (*OsCP4*/*OsCP5*, *OsCP8/OsCP31*, *OsCP20/OCP30*, *OsCP26/OsCP32*, *OsCP31/OsCP32* and *OsVPE2/OsVPE3*) were < 1, suggesting that negative selection on these duplication events occurred (Table 2). In addition, the dates of gene duplication events were figured out as well based on the proposed divergences of rice from other grasses. Gene duplications among *OsCPs* probably occurred from 13.28 to 56.21 million years ago, and gene duplications among *OsVPEs* took place 71.64 million years ago.

**Table 2:** Ka/Ks analysis and duplicated date calculation for *OsCPs* and *OsVPEs*

Duplicated pair	Duplicate type	Ka	Ks	Ka/Ks	Positive selection	Date of gene duplication (million years)
*OsCP4/OsCP5*	Segmental	0.1812	0.1854	0.9773	No	14.26
*OsCP8/OsCP31*	Segmental	0.5674	0.7307	0.7765	No	56.21
*OsCP20/OCP30*	Segmental	0.1456	0.5487	0.2654	No	42.21
*OsCP1/OsCP2*	Tandem	0.3532	0.1727	2.0452	Yes	13.28
*OsCP14/OsCP25*	Tandem	0.3036	0.2298	1.3211	Yes	17.68
*OsCP16/OsCP19*	Tandem	0.3724	0.3459	1.0766	Yes	26.61
*OsCP15/OsCP27*	Tandem	0.3776	0.3423	1.1031	Yes	26.33
*OsCP26/OsCP31*	Tandem	0.4620	0.3929	1.1759	Yes	30.22
*OsCP26/OsCP32*	Tandem	0.3422	0.6900	0.4959	No	53.08
*OsCP31/OsCP32*	Tandem	0.2273	0.4190	0.5425	No	32.23
*OsVPE2/OsVPE3*	Segmental	0.1482	0.9313	0.1591	No	71.64

Ka/Ks < 1 means negative selection, Ka/Ks = 1 means neutral selection, and Ka/Ks > 1 means positive selection

### Protein structure and phylogenetic analysis

**Fig. 3:**
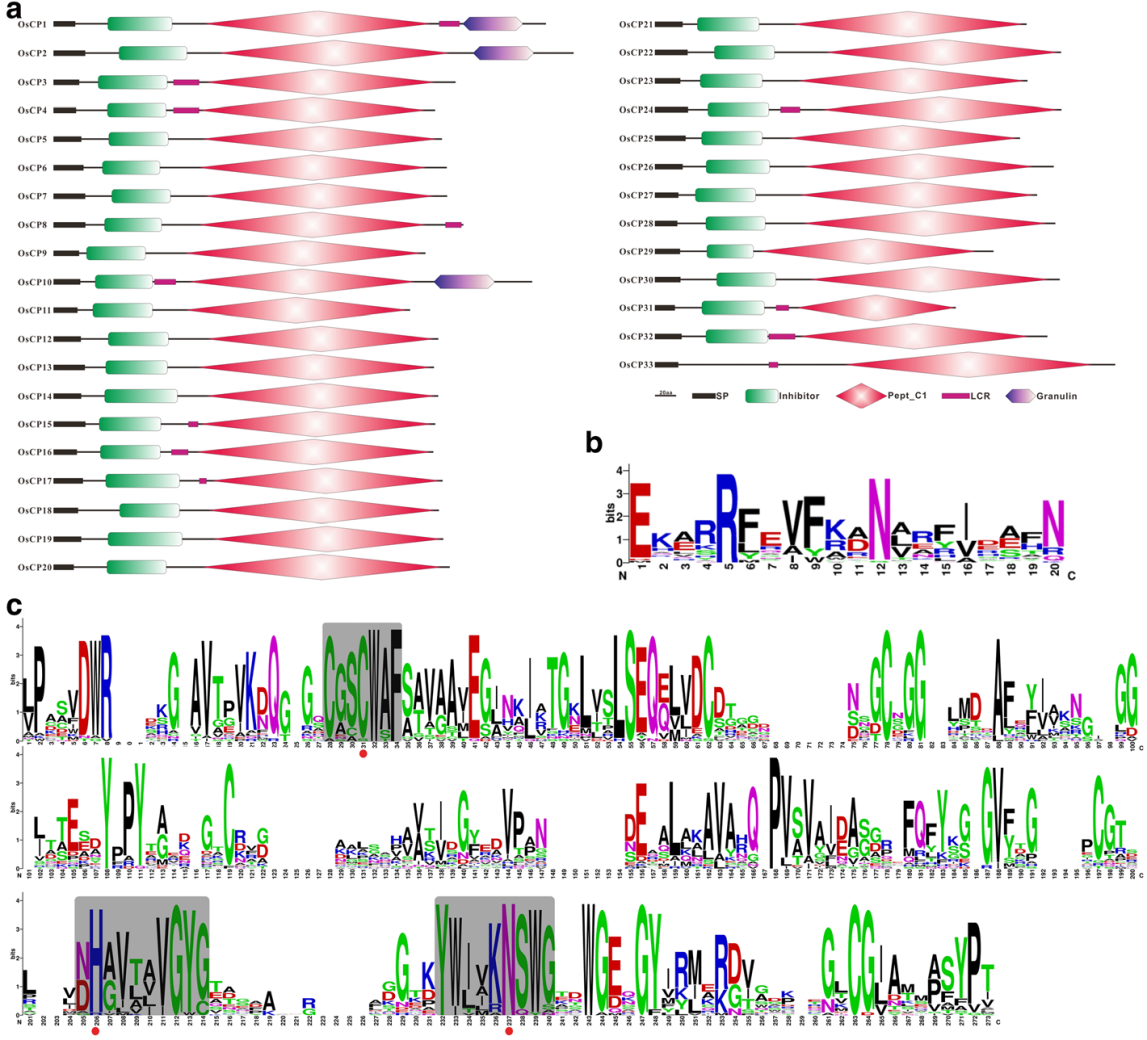
Schematic diagram of OsCPs and analysis of conserved motifs in OsCPs. **a** Schematic diagram of OsCPs. **b** Conservative analysis of the inhibitor domain in OsCPs. **c** Conservative analysis of the peptdiase C1A domain in OsCPs

**Fig. 4:**
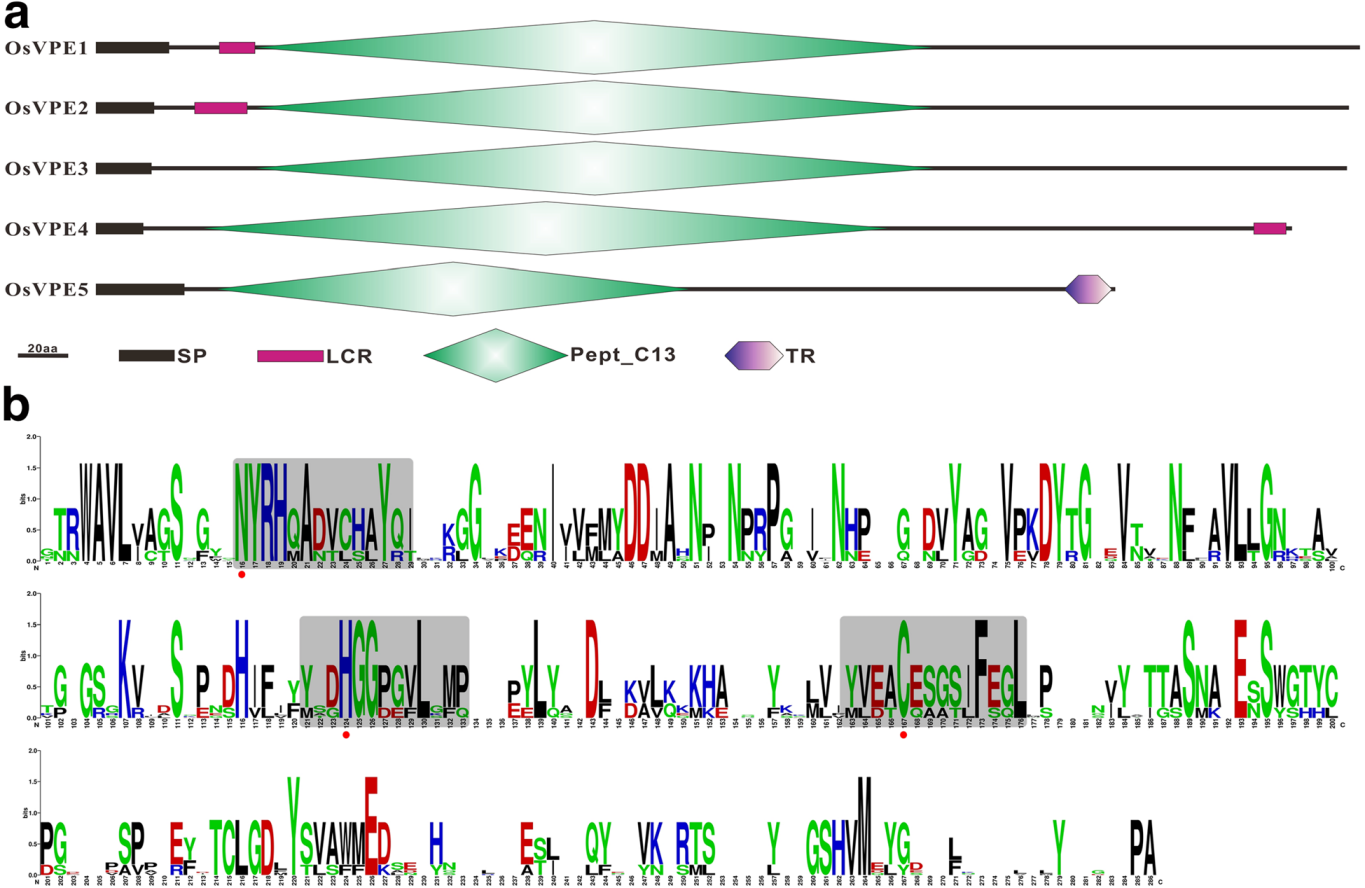
Schematic diagram of OsVPEs and analysis of conserved motifs in OsVPEs. **a** Schematic diagram of OsVPEs. **b** Conservative analysis of peptdiase C13 domain in OsVPEs

To gain insight into potential subcellular location of each OsCP and OsVPE, signal peptide predication of each protein using SignalP 4.1 was firstly carried out [37]. The results revealed that all OsCPs and OsVPEs contain a predicted signal peptide, indicating that all members in these two families could enter the endomembrane system (Figs. 3a and 4a). Subcellular targeting of all members in these two families was also predicted. The results revealed that there are two subcellular targeting sequences in OsCPs. The first is the vacuolar targeting sequence NPIR, which could be detected in the N terminus of OsCP18. The second is the ER targeting sequence, which could be detected both in OsCP3 and OsCP8 (Additional file 1: Figure S1).

Multiple sequence alignment was performed to explore sequence features and to identify functional motifs of two families in rice. As for OsCPs, several typical motifs have been identified. The first, Cathepsin propeptide inhibitor domain was found at the N terminus of all OsCPs except OsCP33 (Fig. 3a and Additional file 1: Figure S1). The motif sequence was ExxxRxxxFxxNxxxI/VxxxN with one mismatch and the most conservative positions in rice are 1, 5 and 12 (Fig. 3b). Instead of “ERFNIN” motif, three OsCPs (OsCP14, OsCP17 and OsCP19) carry a similar “ERWNIR” motif and three OsCPs (OsCP20, OsCP28 and OsCP30) carry a conserved “ERFNAQ” motif just like cathepsin F in animals. Second, catalytic triad (Cys-His-Asn) is conserved in rice PLCPs except OsCP16, in which serine is substituted for cysteine as a nucleophile of enzyme activity, and the amino acids before and after the catalytic triad are also conserved, which could be detected in all OsCPs (Fig. 3c). Third, the active region is highly conserved and rich in polar amino acids. The fourth, a C-terminal extension consisting of a Pro-rich domain followed by a granulin-like domain (Cx_5_Cx_5_CCCx_7_Cx_4_CCx_6_CCx_5_CCx_6_Cx_6_C) was detected in three OsCPs (OsCP1, OsCP2 and OsCP10). Granulins are growth hormones that are released upon wounding in the animal kingdom, but this fusion only occurs in plants and is not detected in animals [5, 38]. However, exact roles of granulin domain in OsCP protein are still waiting to be explored in the further study. In contrast to OsCPs, OsVPEs comprise a shorter N-terminal and a much longer C-terminal propeptide (Fig. 4a and Additional file 2: Figure S2). The active region of OsVPEs, rich in polar amino acids, is highly conserved and the catalytic triad (Cys-His-Asn) is also detected in five OsVPEs (Fig. 4b).

**Fig. 5:**
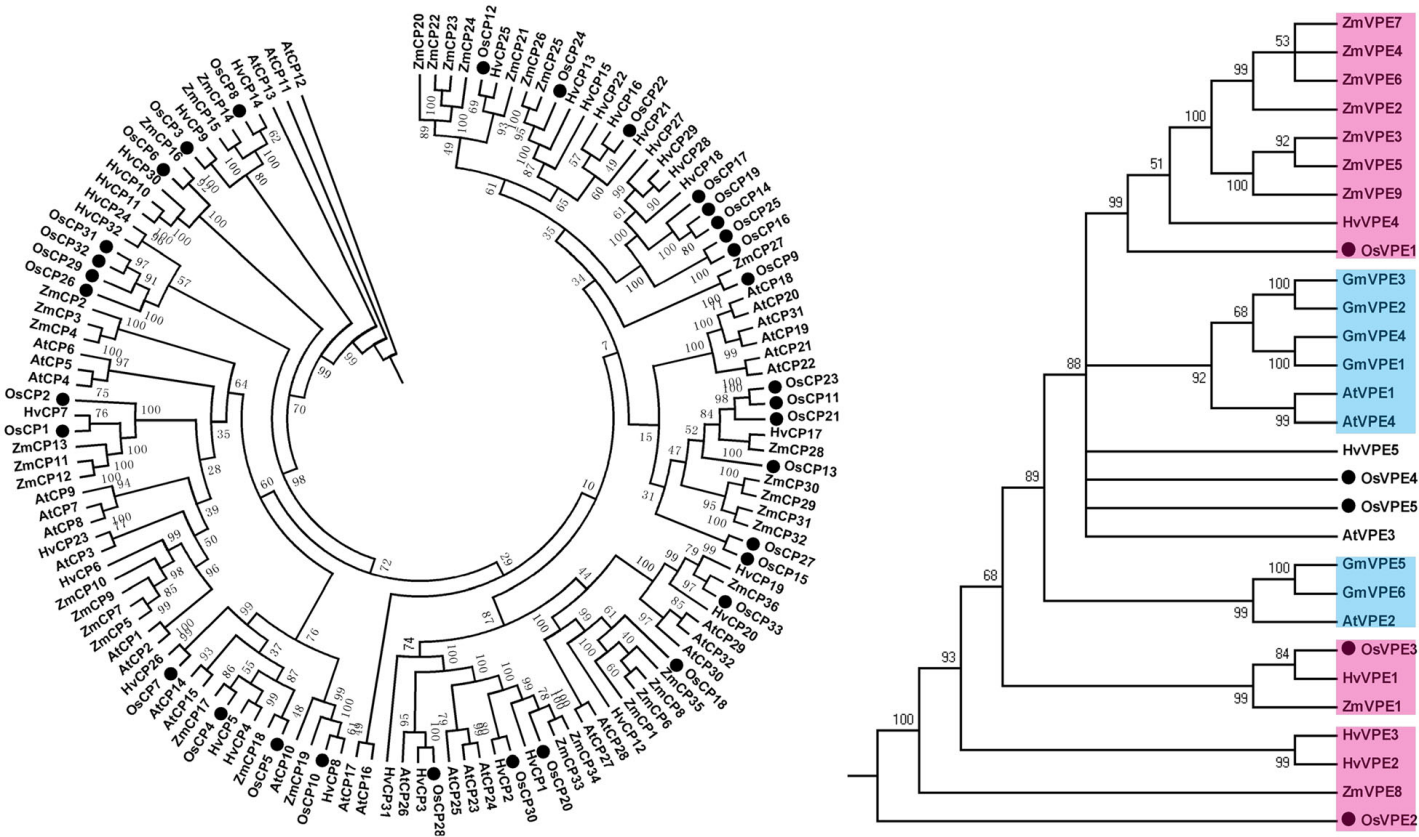
Phylogenetic relationships of papain-like and legumain-like cysteine proteases among rice and other plant species. The tree was calculated with Phylip Ver. 3.68 software using the Protpars method. Rice papain-like and legumain-like cysteine proteases were marked by black dots and plant legumain-like cysteine proteases from monocots and eudicots were shaded in purple and light blue respectively. The numbers at the nodes indicate the bootstrap values

To further analyze phylogenetic relationships of OsCPs and OsVPEs to their counterparts from other plants, a total 133 PLCPs from *Hordeum vulgare*, *Zea mays* and *A. thaliana,* and 29 LLCPs from *H. vulgare*, *Z. mays*, *A. thaliana* and *Glycine max* were used to construct a phylogenetic tree. OsCPs were distributed evenly across evolutionary tree branches. Phylogenetic relationship did not reflect the distinction between monocot and eudicot plants, just like the cystatins in rice (Fig. 5a) [39]. However, LLCPs were divided into the two independent monocots and eudicots, indicating their functional difference between monocots and eudicots LLCPs (Fig. 5b).

### Expression pattern of *OsCPs* and *OsVPE*s in different tissues under normal conditions

To assess the potential functions of *OsCPs* and *OsVPEs* during rice development, their expression pattern was revealed by two approaches: publicly available expression data and real-time reverse transcription-PCR (RT-qPCR). The expression data of two families from the spatio-temporal gene expression profiles of various tissues/organs at RiceXPro (Rice Expression Profile Database) were obtained and summarized to construct expression profile of *OsCPs* and *OsVPEs. OsCPs* display diverse expression patterns as shown in Additional file 3: Figure S3. The transcripts of most *OsCPs* could be detected in the same tissue, for example, at least 20 *OsCPs* were detected in roots, 8 genes in flag leaf, 6 genes in palea and lemma, indicating that rice PLCPs may function redundantly in vivo. Two *OsCPs* (*OsCP6* and *OsCP28*) display a similar expression pattern, which are predominantly expressed in embryo and endosperm, but lower in other tissue tested. Expression pattern of *OsCPs* and *OsVPEs* derived from MPSS (massively parallel signature sequencing) were also listed in Additional file 4: Table S4, which is similar to the results from RiceXPro.

**Fig. 6:**
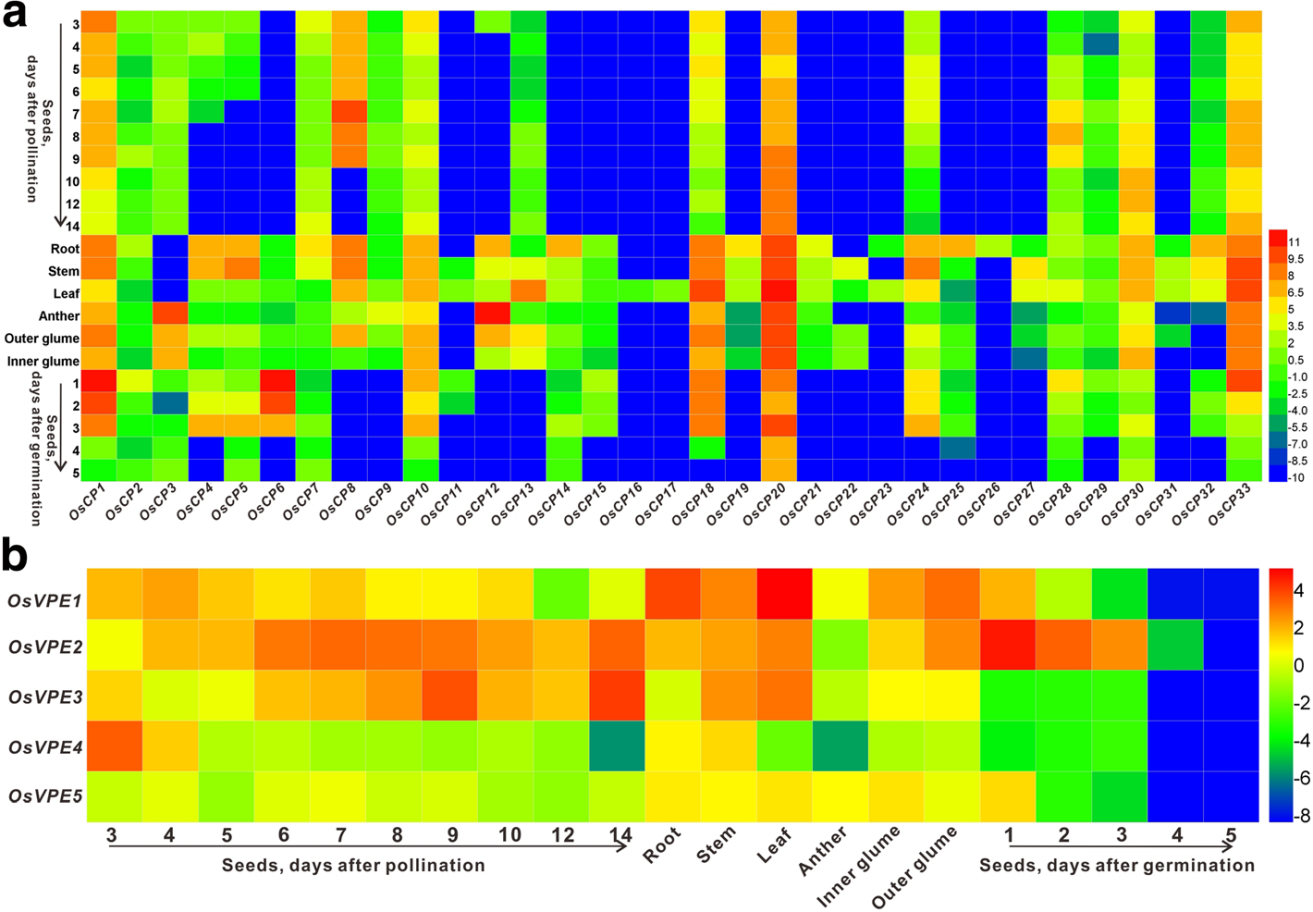
Heatmap analysis of *OsCP* and *OsVPE* gene expression. (**a**). Heatmap analysis of *OsCPs*, (**b**). Heatmap analysis of *OsVPEs*. The expression was normalized against *OsActin* and *OsUBC*, and data represent the mean with three independent experiments. A red box indicates the higher expression level, whereas the blue box indicates the lower expression level. The scale bar represents the fold change (log2 value)

To confirm the public data, RT-qPCR was used to construct the expression profile of *OsCPs* and *OsVPEs*. cDNAs prepared from different tissues such as leaves, stems, roots, anther, out glume, inner glume and seeds at different developmental stages were chosen as templates for RT-qPCR. Similar to the public data, heatmap analysis based on the relative expression level show that most members of *OsCPs* display diverse expression pattern (Fig. 6a). Three *OsCPs* (*OsCP1*, *OsCP20* and *OsCP33*) were abundantly expressed in each tissue tested. Two *OsCPs* (*OsCP3* and *OsCP12*) had a similar expression pattern, both preferentially expressed in anther, indicating a potential role in anther development. Generally, *VPEs* in plants could be separated into two subfamilies: vegetative-type *VPEs* and seed-type *VPEs* [40]. However, *OsVPEs* displayed a rather broad expression profile, which could be detected in both seed and vegetative tissues like *HvLeg-2* and *HvLeg-4* in barley (Fig. 6b and Additional file 5: Figure S5) [41].

### *OsCPs* and *OsVPEs* display dynamic expression pattern during the processes of seed development and germination

**Fig. 7:**
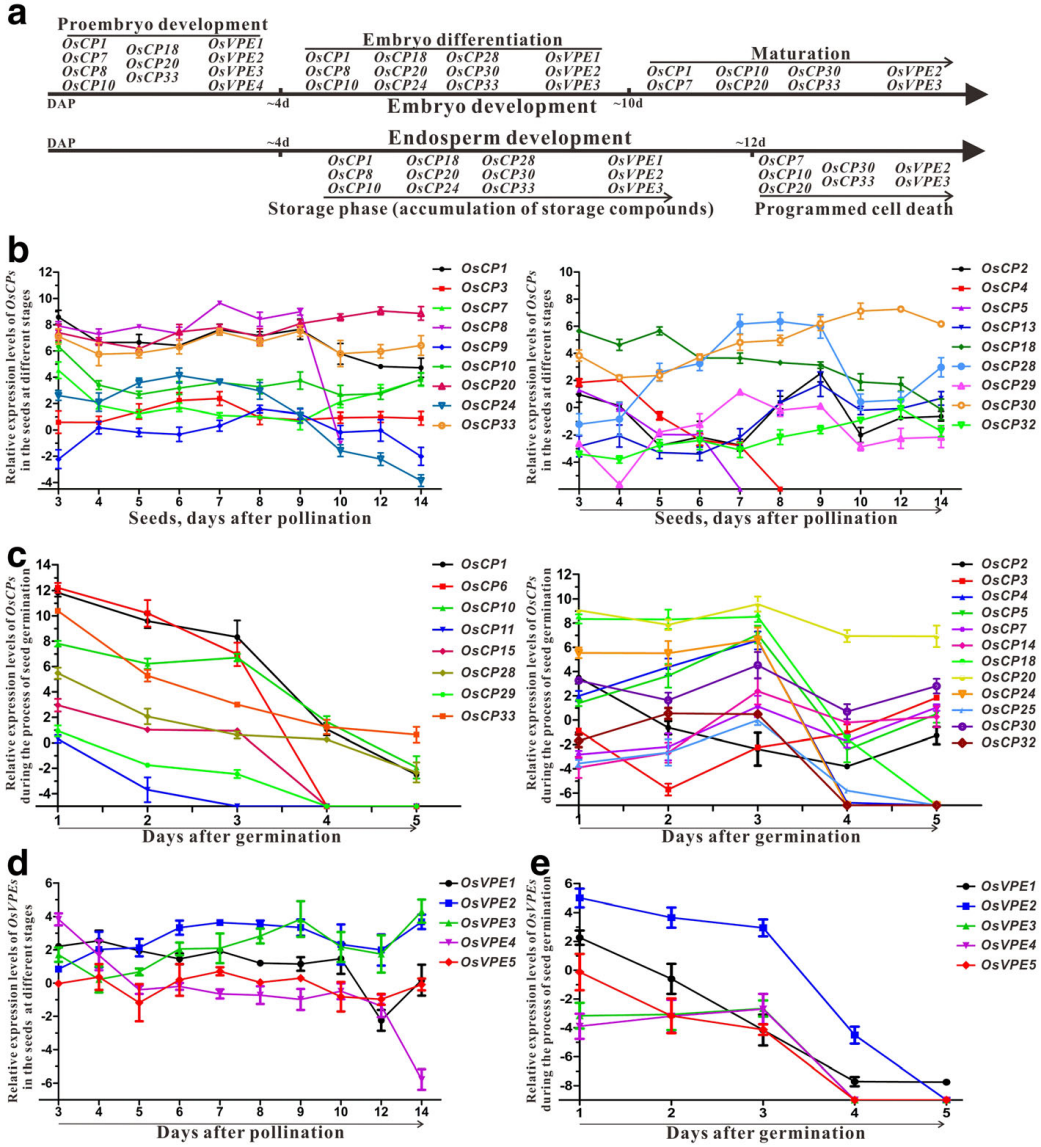
Dynamic changes of the expression level of *OsCPs* and *OsVPEs* during the processes of seed development and germination. (**a**). Overview of the expression of *OsCPs* and *OsVPEs* during the processes of embryo and endosperm development. (**b**-**c**). Dynamic changes of the expression level of *OsCPs* during the processes of seed development (**b**) and germination (**c**). (**d**-**e**). Dynamic changes of the expression level of *OsVPEs* during the processes of seed development (**c**) and germination (**d**). The expression was normalized against *OsActin* and *OsUBC*. The data represent fold change (log2 value) and bars indicate the standard deviation of with three independent repetitions

Early reports demonstrated that both PLCPs and LLCPs are involved in seed formation and seed germination [7, 8, 27, 33, 42]. To explore potential roles of two family genes in the process of rice seed development, the transcriptional level of each gene in seeds at different developmental stages and different germination stages were comprehensively analyzed. In general, more than half of *OsCPs* and all the *OsVPEs* could be detected in seeds at different stages (Fig. 7). Among them, four *OsCP*s (*OsCP1*, *OsCP8*, *OsCP20* and *OsCP33*) and three *OsVPE*s (*OsVPE1*, *OsVPE2* and *OsVPE3*) were abundant in seeds. It is common knowledge that seed development in rice consists of the development of embryo and endosperm and the former could be divided into three stages: proembryo development, embryo differentiation and maturation [43]. Seven *OsCPs* and four *OsVPEs* were abundant in seeds corresponding to the proembryo developmental stage and nine *OsCPs* and three *OsVPEs* could be detected in the seeds (4~ 10 days after pollination, DAP), speculating their roles in organ differentiation of rice embryo development (Fig. 7a). During the process of endosperm development, accumulation of storage compounds is very important and closely related to grain production and quality. Expression pattern analysis revealed that twelve *OsCPs* (*OsCP1*, *OsCP8*, *OsCP10*, *OsCP18*, *OsCP20*, *OsCP24*, *OsCP28*, *OsCP30*, *OsCP33*, *OsVPE1*, *OsVPE2* and *OsVPE3*) were strongly expressed in this stage, indicating that they may take part in the processing of storage proteins during storage phase of endosperm development. After 12 days, endosperm cells begin to degrade through PCD, during which the expression levels of five *OsCPs* and two *OsVPEs* still kept high, suggesting that these genes perhaps participated in the degradation of endosperm cells.

From the view of dynamic change of the expression pattern, *OsCP*s could be grouped into four classes (Fig. 7b). The expression level of the first group is relatively stable and shows no visible change during the whole process of seed formation. Four *OsCP* genes (*OsCP1*, *OsCP3*, *OsCP8* and *OsCP33*) fall into this group. The second group consists of three *OsCP*s (*OsCP4*, *OsCP5* and *OsCP12*) and the transcripts of them could only be detected in the seeds before 7 DAP, suggesting an important role in early seed development. The third group is that their expression peak is at seeds 7–9 DAP. *OsCP2*, *OsCP13*, *OsCP28* and *OsCP29* belong to this group. The last group contains the rest of *OsCP*s whose expression levels show dynamic changes during the process of seed development in rice. As for *OsVPE* genes, the transcriptional level of *OsVPE4* decreases gradually during the processes of seed formation, and the other members exhibited relatively stable expression patterns (Fig. 7d).

In the process of seed germination, most of *OsCPs* and all the *OsVPEs* could be detected and the striking feature is that the expression level of all the members of *OsVPEs* decreased remarkably in the process of seed germination (Fig. 7E). Several *OsCP* genes (*OsCP1*, *OsCP6*, *OsCP10*, *OsCP11*, *OsCP15*, *OsCP28*, *OsCP29* and *OsCP33)* showed similar expression pattern as *OsVPEs* (Fig. 7c). The transcriptional level of the rest *OsCP*s expressed in the germinating seeds varied significantly during the process of seed germination, except *OsCP20,* which showed an abundant and relatively stable expression pattern.

### Differential responses of *OsCP*s to hormone and stress treatments

**Fig. 8:**
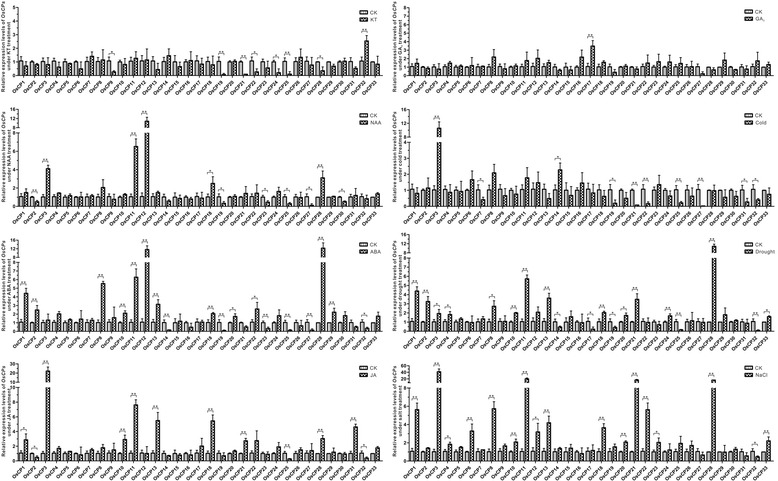
Expression levels of *OsCPs* in seedlings under different hormone and abiotic treatments. The expression was normalized against *OsActin* and *OsUBC*. The data represent the relative expression level compared with that in seedlings under normal growth conditions and bars indicate the standard deviation of with three independent repetitions. ‘*’ and ‘**’ indicate statistical difference compared to the WT (t-test, *p* < 0.05 or 0.01, respectively)

A remarkable feature of PLCPs from plants is that the transcription of them is regulated by different hormones and various stresses [17, 44, 45], and thus function in different physiological processes. To gain insight into their potential roles in response to various hormones and different severe environments, their relative transcriptional levels in seedlings after different hormones treatments (NAA, KT, ABA, GA_3_ and JA) and abiotic treatments (cold, drought and salt) were investigated by RT-qPCR. Based on the relative expression level of each *OsCP*, histograms were created (Fig. 8) and overview of *OsCPs* in response to different hormones and abiotic stresses was listed in Table 3.

**Table 3:** Overview of *OsCPs* and *OsVPEs* in response to different hormones and abiotic stresses

Name	Hormone					Abiotic stress		
	KT	NAA	ABA	GA	JA	Cold	Drought	Salt
*OsCP1*	No	No	++	No	+	No	++	++
*OsCP2*	No	–	+	No	–	No	+	No
*OsCP3*	No	++	No	No	++++	+++	No	+++++
*OsCP4*	No	No	No	No	No	No	No	No
*OsCP5*	No	No	No	No	No	No	No	No
*OsCP6*	No	No	No	No	No	No	No	+
*OsCP7*	No	No	No	No	No	–	No	No
*OsCP8*	No	No	++	No	No	No	+	++
*OsCP9*	–	No	No	No	No	No	No	No
*OsCP10*	No	No	+	No	+	No	+	+
*OsCP11*	No	++	++	No	++	No	++	+++
*OsCP12*	No	+++	+++	No	No	No	No	+
*OsCP13*	No	No	+	No	++	No	+	++
*OsCP14*	No	No	–	No	No	+	–	No
*OsCP15*	No	No	No	No	No	No	No	No
*OsCP16*	No	No	No	No	No	No	No	No
*OsCP17*	No	No	No	+	No	No	–	No
*OsCP18*	No	+	+	No	++	No	+	+
*OsCP19*	–	–	–	No	No	–	–	No
*OsCP20*	No	No	No	No	No	No	No	+
*OsCP21*	–	No	No	No	+	–	+	++++
*OsCP22*	–	No	+	No	No	–	No	++
*OsCP23*	No	–	–	No	No	No	No	+
*OsCP24*	–	No	No	No	No	No	No	No
*OsCP25*	–	–	–	No	–	–	–	No
*OsCP26*	No	No	No	No	No	No	No	No
*OsCP27*	No	–	–	No	No	–	No	No
*OsCP28*	–	+	+++	No	+	No	+++	++++
*OsCP29*	No	No	+	No	No	No	No	No
*OsCP30*	No	–	No	No	No	No	No	No
*OsCP31*	No	No	No	No	++	–	No	No
*OsCP32*	+	No	–	No	–	–	–	–
*OsCP33*	No	No	No	No	No	No	No	+
*OsVPE1*	No	No	+	No	No	No	+	++
*OsVPE2*	No	No	+	No	+	No	+	++
*OsVPE3*	No	+	++++	No	+	No	++	++++
*OsVPE4*	No	No	No	No	–	No	No	No
*OsVPE5*	No	No	No	No	No	No	No	No

The expression was normalized against *OsActin* and *OsUBC* and data represent the mean with three independent experiments. ‘+’ or ‘-’ represents that the expression level was up-regulated or down-regulated respectively. One ‘+’ or ‘-’ means > 2 fold change; two ‘+’ or ‘-’ mean > 4 fold change; three ‘+’ or ‘-’ mean > 8 fold change; four ‘+’ or ‘-’ mean > 16 fold change; five ‘+’ or ‘-’ mean > 32 fold change

Twenty four *OsCPs* except *OsCP4*, *OsCP5*, *OsCP6*, *OsCP7*, *OsCP15*, *OsCP16*, *OsCP20*, *OsCP26* and *OsCP33* are response to at least one hormone treatment (Table 3 and Fig. 8). However no *OsCP* was commonly regulated by five hormones tested. Generally, these *OsCPs* display variable responses to different stresses. Only *OsCP17* is responsible to GA_3._ After KT treatment, the expression levels of *OsCPs* were commonly down-regulated significantly (< 2 fold) apart from *OsCP32*. For the other three hormone treatments, *OsCPs* exhibited differential expression pattern. The expression level of *OsCP3* (> 16 fold) increased significantly after JA treatment. Whereas the expression level of *OsCP27* (< 16 fold) decreased significantly after NAA treatment. Microarray data of 33 *OsCPs* in 7-day-old seedlings subjected to six hormones (ABA, GA_3_, Auxin, Brassinosteroid, Cytokinin and JA) were also extracted from the Rice Expression Profile Database (Additional file 6: Figure S6). Consistent with our results, *OsCPs* were more sensitive to ABA and JA among six plant hormone treatments.

Apart from nine *OsCP* genes (*OsCP4*, *OsCP5*, *OsCP9*, *OsCP15*, *OsCP16*, *OsCP24*, *OsCP26*, *OsCP29* and *OsCP30*), other *OsCPs* are responsive to different stress treatments (Table 3 and Fig. 8). However, only two *OsCP*s (*OsCP21* and *OsCP32*) show response to all three stresses (> 2 fold). *OsCP32* was always down-regulated by three different stress treatments, which indicated a common role of *OsCP32* in cold, drought and salt stress resistance. However other *OsCP*s were differentially regulated by different stress. In responsible to cold treatment, two *OsCPs* (*OsCP3* and *OsCP14*) were up-regulated (> 2-fold change) and eight *OsCPs* (*OsCP7*, *OsCP19*, *OsCP21*, *OsCP22*, *OsCP25*, *OsCP27*, *OsCP31* and *OsCP32*) were down-regulated (> 2-fold change). Notably, for rice PLCPs, the degree of response to cold stress varied significantly. In response to drought treatment, nine *OsCP*s (*OsCP1*, *OsCP2*, *OsCP8*, *OsCP10*, *OsCP11*, *OsCP13*, *OsCP18*, *OsCP21* and *OsCP28*) were up-regulated and five *OsCP*s (*OsCP14*, *OsCP17*, *OsCP19*, *OsCP25* and *OsCP32*) were down-regulated. In responsible to salt treatment, almost half of *OsCPs* were up-regulated and only *OsCP32* was down-regulated. The expression data of *OsCP*s from MPSS database under abiotic stress treatments were summarized in Additional file 7: Table S7. Three *OsCPs* (*OsCP1*, *OsCP8* and *OsCP25*) showed a similar response to drought stress and the expression level of *OsCP1* was up-regulated after salt treatment which was also detected in present study. Apart from this, potential binding motifs in the promoters of these proteases have been screened, and the results were listed in Additional file 8: Table S8.

### Differential responses of *OsVPE*s to hormone and stress treatments

**Fig. 9:**
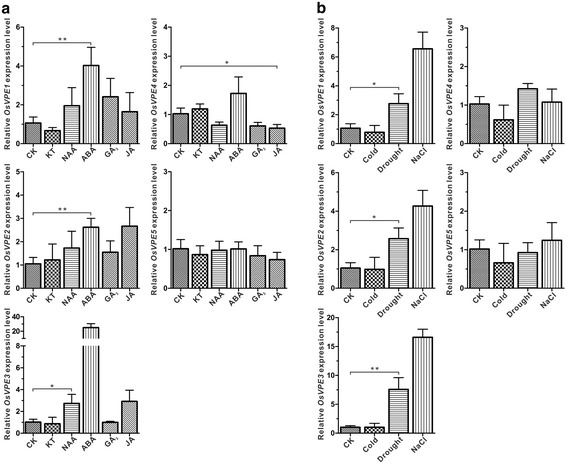
Expression levels of *OsVPEs* in seedlings under different hormone and abiotic treatments. The expression was normalized against *OsActin* and *OsUBC*. The data represent the relative expression level compared with that in seedlings under normal growth conditions and bars indicate the standard deviation of with three independent repetitions. ‘*’ and ‘**’ indicate statistical difference compared to the WT (t-test, *p* < 0.05 or 0.01, respectively)

Similar to PLCPs, the expression level of *VPEs* in plants also increased in the process of senescence [46], wounding [47], pathogen infection [26, 28] and abiotic stresses [46, 47]. To verify whether *VPEs* in rice display similar responses, the relative expression level of each *OsVPE* was quantified after different treatments. Generally, all *OsVPE*s except *OsVPE5* are response to one or several plant hormones or abiotic stresses (Table 3 and Fig. 9). However, different treatments have diverse effects on the change of expression levels of *OsVPE*s. For hormone treatment, no *OsVPE* show response to KT and GA_3._ Only one *OsVPE3* was up-regulated by NAA treatment (Fig. 9a). In contrast, the expression of most *OsVPEs* was regulated by ABA and JA. The expression level of three *OsVPEs* (*OsVPE1*, *OsVPE2* and *OsVPE3*) increased significantly in seedlings after ABA treatment. JA has the same effect on *OsVPE2* and *OsVPE3*, but reverse effect on *OsVPE4* expression. For stress treatments, no *OsVPE* shows response to cold treatment. However, three *OsVPEs* (*OsVPE1*, *OsVPE2* and *OsVPE3*) were commonly regulated (> 2 fold change) by salt and drought treatment, indicating their common roles in tolerance to salt and drought stresses (Fig. 9b).

## Discussion

### Characteristics of the PLCPs in rice

As described above, several typical conserved motifs for PLCPs have been identified in most OsCPs. The catalytic triad (Cys-His-Asn) is essentially responsible for proteolytic activity of PLCPs [48], and this central typical motif could be detected in all of OsCPs except OsCP16, in which serine is substituted for cysteine. “ERFNIN” motif provides the core structure of the auto-inhibitory prodomain in most rice PLCPs [49] and the other OsCPs carry the similar ERFNAQ or ERWNIR motif instead. Generally, PLCPs could be divided into four major groups: cathepsin B-, F-, H-, and L-like proteases according to the motif in N-terminal pre-domain and their closest counterparts in animals and ERFNIN motif is typical for cathepsin L- and H-like proteases, but not for cathepsin B-like proteases [50, 51]. ERFNAQ motif is a marker motif for cathepsin F-like proteases [50]. According to this principle, one cysteine protease OsCP33 was grouped into cathepsin B-like protease, since no typical motif could be detected in pre-domain. Three OsCPs (OsCP20, OsCP28 and OsCP30) with an ERFNAQ motif in their proregions fall into cathepsin F-like protease. And typical ERFNIN motif could be detected in the remaining OsCPs through the alignment of protein sequences. However, differences in their substrates and physiological roles of papain-like proteases in different groups are still largely unknown. Granulin-like domain (Cx_5_Cx_5_CCCx_7_Cx_4_CCx_6_CCx_5_CCx_6_Cx_6_C), which may serve to regulate thiolprotease activity in plants, was also detected in the C-terminal of several rice PLCPs (OsCP1, OsCP2 and OsCP10). Although the fusion of a granulin domain in the C-terminal of PLCPs has been observed in several plants, but the exact roles of this domain in PLCPs needs to be further studied in the future.

### Potential roles of *OsCPs* in seed development

During past decades, PLCPs were reported to play essential roles in different developmental processes, especially in various types of PCD in different tissues. NtCP14, a papain-like protease with a granulin domain in the C terminal, was approved as a key protease in triggering PCD of suspensor in early embryogenesis. Overexpression of *NtCP14* could induce precocious cell death of basal cell lineages of the embryo [52]. Besides its role in PCD of suspensor cell, PLCPs were also associated with the development of inner integument. In *Brassica napus*, *BnCysP1* encoding a papain-like protease was reported to be responsible for PCD of the inner integument [53]. In the present study, our expression pattern analysis results revealed that the transcripts of all *OsCPs* could be detected in seeds, but the expression levels of them display dynamic changes during the process of seed development in rice, indicating potential roles of PLCPs in seed development, potentially in PCD of endosperm, which are worthy to be explored in the future study. Another striking feature of the expression profile is that the expression level of most *OsCPs* display striking changes during the process of seed germination, indicating potential roles in regulating seed germination. In barley, two cathepsin L-like proteases, HvPap-6 and HvPap-10, could degrade B, C, and D hordeins stored in the endosperm of barley seeds, which is critical for successful seed germination [7]. Similarly, a gibberellin-inducible cysteine proteinase named gliadain, could digest the storage protein gliadin into low molecular mass peptide almost specifically in wheat for seed germination [8].

There are also some examples indicating that PLCPs are regulated not only at the transcriptional level, but also at the level of protease activity. In barley, the transcripts of a cathepsin B-like cysteine protease (*CatB*) increased upon germination in the aleurone, leading to the increase of *CatB* activities in the process of seed germination [44]. Similarly, cathepsin L-like peptidases have also been shown to be involved in the mobilization of hordeins in the barley seed but this process could be partially inhibited by barley cystatins [7]. In the present study, the transcripts of four *OsCPs* (*OsCP1*, *OsCP6*, *OsCP18* and *OsCP20*) are abundant during the first three days of seed germination and decreased later. Consistent with this result, the expression levels of most cystatin genes were higher in seeds at early stages and then decreased dramatically upon seed germination [39]. Hence, the balance between cystatins and PLCPs seems important for seed germination.

### Relationship between papain-like and legumain-like cysteine proteases

Papain-like and LLCPs are two important proteolytic enzymes in two subfamilies of cysteine proteases in the Merops protease database [1], which are usually synthesized as inactive poenzyme and use a catalytic Cys as a nucleophile during proteolysis. Auto-inhibitory domain in the N-terminal of PLCPs will be processed to generate a mature form in an acid condition [54]. In contrast, autocatalytic activation of the LLCPs needs two sequential steps by cleavage of the C- and N-terminal propeptides [23]. Although many distinctions between two families in protein structure and biochemical properties exist, the activities of both of them could be inhibited by a group of small proteins called cystatins [2, 3], which spontaneously raises questions about whether papain-like and LLCPs are cross-linked in same biological process.

Previous work has proved that both papain-like and LLCPs participated in hypersensitive response (HR) [15, 26, 28] and seed germination [7, 8, 19]. During the hypersensitive response (HR), the transcriptional level of a papain-like protease called *NbCathB* was quickly induced, which is critical for HR. When the activities was blocked by treatment with protease inhibitors or downregulation of *NbCathB*, the HR induced by two distinct nonhost bacterial pathogens (*Erwinia amylovora* and *Pseudomonas syringae* pv. *Tomato*) was prevented [15]. Similarly roles of LLCPs in HR have also been found. Silencing of *VPEs* in *N. benthamiana* could abolish the hypersensitive cell death triggered by tobacco mosaic virus (TMV) [26]. In addition, both papain-like and LLCPs are presumed to be responsible for the mobilization of the storage proteins during the process of seed germination. The storage protein-phaseolin in common bean could not be degraded either by papain-like protease CPPh1 or by legumain-like proteases LLP, but only be degraded by papain-like protease CPPh1 and legumain-like proteases LLP in a synergetic way [55]. Furthermore, VmPE-1 had a potential to process the papain-like proteinase designated SH-EP to its intermediate in vitro, which had a major role in the degradation of seed storage protein in *Vigna mungo* [42]. All these data implied that papain-like and LLCPs may be linked together in many physiological processes. In the present study, some papain-like and legumain-like family genes were found to have a similar expression pattern, for example, *OsCP10*/*OsCP18*/*OsVPE4* during the process of seed formation and *OsCP1*/*OsCP6*/*OsVPE2* during the process of seed germination. Furthermore, the expression of some papain-like and legumain-like family genes (*OsCP8*/*OsVPE1*, *OsCP1*/*OsCP13*/*OsVPE2* and *OsCP11*/*OsCP18*/*OsCP28*/*OsVPE3*) are commonly regulated by hormones and different abiotic stresses, suggesting their potential cooperative roles in plant development and stress environments.

## Conclusions

In the present study, 33 *OsCPs* encoding PLCPs and 5 *OsVPEs* encoding LLCPs were identified in rice genome respectively. Systematic analysis of *OsCP* and *OsVPE* family genes including gene structure, chromosomal distribution, gene duplication, phylogenetic relationship, sequence characteristics and expression pattern analysis were performed. Comprehensive expression profile analysis of both families during the whole process of seed development and germination was also carried out, suggesting their potential roles in seed development and germination. RT-qPCR analysis during diverse stress environments revealed that most of them were regulated by plant hormones and in response to different stress treatments including cold, drought and salt stress. This work suggests their common roles in seed development and stress tolerance, which provides potential clues for further in-depth study of the selected genes in two families.

## Methods

### Identification of *OsCPs* and *OsVPEs* in rice genome

To identify *OsCPs* and *OsVPEs* in *O. sativa*, tBLASTP program of the National Center for Biotechnology Information (NCBI) in the rice protein database (http://www.ncbi.nlm.nih.gov/) with AtCP and AtVPE protein sequences of *A. thaliana* was performed. Returned nucleotide sequences were considered as *OsCP* and *OsVPE* candidates. After removing the redundant genes, deduced protein sequences of all putative OsCP and OsVPE were used to perform BLASTP program, and the sequences with intact peptidase C1A and peptidase C13 domain were considered as true *OsCPs* and *OsVPEs* in *O. sativa*. Corresponding full-length cDNAs were downloaded from Rice Functional Genomic Express Database (http://signal.salk.edu/cgi-bin/RiceGE).

### Analysis of genomic structure and chromosomal localization

Exon-intron organization was determined by the alignment of their coding sequence to their corresponding genome full-length sequence. Diagrams were drawn with Gene Structure Display Server (GSDS: http://gsds.cbi.pku.edu.cn/). *OsCPs* and *OsVPEs* were positioned on the rice chromosomes using BLASTN at the Rice Genome Annotation Project website (http://rice.plantbiology.msu.edu/).

### Gene duplication and duplication date calculation

Genes on the duplicated chromosomal segments were identified using Plant Genome Duplication Database (http://chibba.agtec.uga.edu/duplication/) with the maximum distance permitted between collinear gene pairs of 500 kb. Homologous genes separated by five genes at most were regarded as tandem duplicated genes. Calculation of the duplication dates was according to the previous methods [39].

### Protein sequence alignment and phylogenetic analysis

Multiple sequence alignments of amino acid sequences were performed using Clustal X ver. 1.81 with the default multiple alignment parameters. Phylogenetic tree was generated with Phylip Ver. 3.68 using the Protpars method. Protein sequences of papain-like and LLCPs from *A. thaliana*, *H. vulgare* and *Z. mays* were used in this analysis, and their accession numbers are listed in Additional file 9: Table S9 and Additional file 10: Table S10.

### Digital expression analysis of *OsCPs* and *OsVPEs*

Expression profile data from rice microarrays are available in the Rice Expression Profile Database (http://ricexpro.dna.affrc.go.jp), which is a repository of gene expression profiles derived from microarray analysis of tissues/organs encompassing the entire life cycle of the rice plant under natural and plant hormone-treated conditions. The expression results of *OsCPs* and *OsVPEs* were summarized in Additional file 6: Figure S6 and Additional file 11: Figure S11.

Rice MPSS database (http://mpss.udel.edu/rice/mpss_index.php) was searched to obtain the expression levels of *OsCPs* and *OsVPEs*. The criteria was that the signature must be unique in the genome (hits = 1) and a perfect match (100% identity over the tag length). TPM (tags per million) means the normalized abundance of the signatures, which is the best estimate of the expression level for a given gene. The expression data under normal conditions were listed in Additional file 4: Table S4. The expression data from rice plants under abiotic stress treatments were summarized in Additional file 7: Table S7.

### Plant materials and the methods for various treatments

*Oryza sativa L. japonica cv. Nipponbare* was grown in the greenhouse at Wuhan University with temperature difference between day and night (30/26 °C) under a photoperiod of 16 h light and 8 h dark. For the expression pattern analyses of *OsCPs* and *OsVPEs* under normal conditions, total RNA was extracted from root, stem and leaf of 5-day-old seedlings growing on 1/2 MS solid medium and other different tissues including anther, out glume, inner glume, seeds at different developmental stages.

For various treatments, seeds were sterilized according to previous protocol [39] and then inoculated on 1/2 MS solid medium contain 1% sucrose. For Abscisic acid (ABA), Gibberellin acid (GA_3_), 1-Naphthaleneacetic acid (NAA), Kinetin (KT), Jasmonic acid (JA) treatment, 5-day-old seedlings were cultured in 1/2 MS liquid medium containing 25 μM ABA, 5 μM GA_3_, 5 μΜ NAA, 5 μM KT and 50 μM JA for 12 h, respectively [35]. For salt stress, 5-day-old seedlings were transferred into 1/2 MS liquid medium containing 400 mM NaCl; for drought stress, seedlings were dried between folds of the sterile filter paper; for cold stress, the culture plates containing the seedlings were kept at 4 °C. All the treatments were for 12 h.

### cDNA synthesis and RT-qPCR

Total RNA of different tissues were isolated with Trizol reagent according to the manufacturer’s instructions (Life Technology,USA). The residual genomic DNA was removed by RNase-free DNase I (Promega, USA). First-strand cDNA was synthesized using M-MLV reverse transcriptase following the manufacturer’s instructions (Invitrogen, USA). RT-qPCR was introduced for *OsCPs* and *OsVPEs* expression analysis according to the protocol described previously [56]. Five house-keeping genes including *ACTIN*, *eEF-1a*, *UBC*, *UBQ5* and *GAPDH* were chosen as internal reference genes for RT-qPCR. The stability of five reference genes in different tissues was evaluated using geNorm (Version 3.5). Two most stable reference genes *ACTIN* and *UBC* were chosen for the calculating normalization factors for different tissues. Thus, the relative expression level of each gene in different tissues was calculated according to the previous protocol [56]. Gene-specific primers were listed in Additional file 12: Table S12.

## Additional files

Additional file 1: **Figure S1.** Multiple protein sequences alignment of rice papain-like cysteine proteases. The inhibitor domain and peptidase C1A domain was shaded in red and black respectively. The granulin domain was marked with black boxes. The similar amino acid residues were marked in blue and the identical acid residues were boxed in purple. Red and black dots indicated the catalytic triad and the retention signal in ER respectively. The ‘NPIR’ was shaded in a black ellipse. (DOCX 868 kb)

Additional file 2: **Figure S2.** Multiple protein sequences alignment of rice legumain-like cysteine proteases. The peptidase C13 domain was shaded. The similar amino acid residues were marked in blue and the identical acid residues were boxed in purple. Red dots indicate the catalytic triad. (DOCX 481 kb)

Additional file 3: **Figure S3.** Expression heatmap of *OsCPs* in different tissues under normal conditions. (DOCX 2617 kb)

Additional file 4: **Table S4.** Expression abundance of *OsCPs* and *OsVPEs* in various tissues under normal conditions. (DOCX 20 kb)

Additional file 5: **Figure S5.** Expression heatmap of *OsVPEs* in different tissues under normal conditions. (DOCX 263 kb)

Additional file 6: **Figure S6.** Expression profile of *OsCPs* in the shoots and roots under different plant hormones treatments. (DOCX 482 kb)

Additional file 7: **Table S7.** Expression change of *OsCPs* and *OsVPEs* under stress treatments**.** The date was from MPSS: gene analysis (http://mpss.udel.edu/rice/GeneQuery.php). The experiment materials were 14-day-old seedlings. Salt treatment: 250 mM NACL for 24 h; drought treatment: stressed in drought for 5d; cold treatment: 4 °C for 24 h. Compared with normal condition, the expression fold change > 2 or < 0.5 were indicated in red or blue respectively. (DOCX 16 kb)

Additional file 8: **Table S8.** Overview of cis-elements in the promoters of *OsCPs* and *OsVPEs*. ABRE: ABA-responsive element; LTRE: Low-temperature-responsive element; DRE: Dethydration-responsive-element; T/G-box: DNA-binding motif of MYC2 (the key transcriptional activator of jasmonate responses). The number of predicted cis-elements were presented by the number of “+”. (DOCX 17 kb)

Additional file 9: **Table S9.** Papain-like Cysteine Proteases in three plant species. (DOCX 18 kb)

Additional file 10: **Table S10.** Legumain-like Cysteine Proteases in four plant species. (DOCX 16 kb)

Additional file 11: **Figure S11.** Expression profile of *OsVPEs* in the shoots and roots under different plant hormones treatments. (DOCX 214 kb)

Additional file 12: **Table S12.** Primers used in this study. (DOCX 2617 kb)

## Abbreviations

ABA: Abscisic acid
GA_3_: Gibberellin acid
HR: Hypersensitive response
JA: Jasmonic acid
Ka: Nonsynonymous
Ks: Synonymous
KT: Kinetin
LLCPs: Legumain-like cysteine proteases
MPSS: Massively parallel signature sequencing
NAA: 1-Naphthaleneacetic acid
PCD: Programmed cell death
PLCPs: Papain-like cysteine proteases
RiceXPro: Rice Expression Profile Database
RT–qPCR: Quantitative real-time reverse transcription-PCR
VIGS: Virus-induced gene silencing
VPE: Vacuolar processing enzyme

## References

[1] Rawlings ND, Barrett AJ, Bateman A (2010). MEROPS: the peptidase database. Nucleic Acids Res.

[2] Arai S, Watanabe H, Kondo H, Emori Y, Abe K (1991). Papain-inhibitory activity of oryzacystatin, a rice seed cysteine proteinase inhibitor, depends on the central Gln-Val-Val-ala-Gly region conserved among cystatin superfamily members. J Biochem.

[3] Alvarez-Fernandez M, Barrett AJ, Gerhartz B, Dando PM, Ni J, Abrahamson M (1999). Inhibition of mammalian legumain by some cystatins is due to a novel second reactive site. J Biol Chem.

[4] Turk V, Turk B, Turk D (2001). Lysosomal cysteine proteases: facts and opportunities. EMBO J.

[5] Richau KH, Kaschani F, Verdoes M, Pansuriya TC, Niessen S, Stuber K, Colby T, Overkleeft HS, Bogyo M, Van der Hoorn RA (2012). Subclassification and biochemical analysis of plant papain-like cysteine proteases displays subfamily-specific characteristics. Plant Physiol.

[6] Wiederanders B, Kaulmann G, Schilling K (2003). Functions of propeptide parts in cysteine proteases. Curr Protein Pept Sc.

[7] Martinez M, Cambra I, Carrillo L, Diaz-Mendoza M, Diaz I (2009). Characterization of the entire cystatin gene family in barley and their target cathepsin L-like cysteine-proteases, partners in the hordein mobilization during seed germination. Plant Physiol.

[8] Kiyosaki T, Matsumoto I, Asakura T, Funaki J, Kuroda M, Misaka T, Arai S, Abe K (2007). Gliadain, a gibberellin-inducible cysteine proteinase occurring in germinating seeds of wheat, *Triticum aestivum* L., specifically digests gliadin and is regulated by intrinsic cystatins. FEBS J.

[9] Zhang XM, Wang Y, Lv XM, Li H, Sun P, Lu H, Li FL (2009). NtCP56, a new cysteine protease in *Nicotiana tabacum* L., involved in pollen grain development. J Exp Bot.

[10] Zhang D, Liu D, Lv X, Wang Y, Xun Z, Liu Z, Li F, Lu H (2014). The cysteine protease CEP1, a key executor involved in tapetal programmed cell death, regulates pollen development in *Arabidopsis*. Plant Cell.

[11] Li N, Zhang DS, Liu HS, Yin CS, Li XX, Liang WQ, Yuan Z, Xu B, Chu HW, Wang J (2006). The rice tapetum degeneration retardation gene is required for tapetum degradation and anther development. Plant Cell.

[12] Lee S, Jung KH, An G, Chung YY (2004). Isolation and characterization of a rice cysteine protease gene, *OsCP1*, using T-DNA gene-trap system. Plant Mol Biol.

[13] Ueda T, Seo S, Ohashi Y, Hashimoto J (2000). Circadian and senescence-enhanced expression of a tobacco cysteine protease gene. Plant Mol Biol.

[14] Kruger J, Thomas CM, Golstein C, Dixon MS, Smoker M, Tang S, Mulder L, Jones JD (2002). A tomato cysteine protease required for *Cf-2*-dependent disease resistance and suppression of autonecrosis. Science.

[15] Gilroy EM, Hein I, van der Hoorn R, Boevink PC, Venter E, McLellan H, Kaffarnik F, Hrubikova K, Shaw J, Holeva M (2007). Involvement of cathepsin B in the plant disease resistance hypersensitive response. Plant J.

[16] Pechan T, Ye L, Chang Y, Mitra A, Lin L, Davis FM, Williams WP, Luthe DS (2000). A unique 33-kD cysteine proteinase accumulates in response to larval feeding in maize genotypes resistant to fall armyworm and other Lepidoptera. Plant Cell.

[17] Khanna-Chopra R, Srivalli B, Ahlawat YS (1999). Drought induces many forms of cysteine proteases not observed during natural senescence. Biochem Bioph Res Co.

[18] Hiraiwa N, Nishimura M, Hara-Nishimura I (1999). Vacuolar processing enzyme is self-catalytically activated by sequential removal of the C-terminal and N-terminal propeptides. FEBS Lett.

[19] Hara-Nishimura I, Inoue K, Nishimura M (1991). A unique vacuolar processing enzyme responsible for conversion of several proprotein precursors into the mature forms. FEBS Lett.

[20] Hara-Nishimura I (1995). Vacuolar processing enzyme responsible for maturation of vacuolar proteins. Seikagaku. The Journal of Japanese Biochemical Society.

[21] Muntz K, Shutov AD. Legumains and their functions in plants. Trends Plant Sci 2002; 7(8):340-344. 10.1016/s1360-1385(02)02298-7 12167328

[22] Hara-Nishimura I, Takeuchi Y, Nishimura M (1993). Molecular characterization of a vacuolar processing enzyme related to a putative cysteine proteinase of *Schistosoma mansoni*. Plant Cell.

[23] Kuroyanagi M, Nishimura M, Hara-Nishimura I (2002). Activation of Arabidopsis vacuolar processing enzyme by self-catalytic removal of an auto-inhibitory domain of the C-terminal propeptide. Plant Cell Physiol.

[24] Shimada T, Yamada K, Kataoka M, Nakaune S, Koumoto Y, Kuroyanagi M, Tabata S, Kato T, Shinozaki K, Seki M (2003). Vacuolar processing enzymes are essential for proper processing of seed storage proteins in *Arabidopsis thaliana*. J Biol Chem.

[25] Hatsugai N, Kuroyanagi M, Nishimura M, Hara-Nishimura I (2006). A cellular suicide strategy of plants: vacuole-mediated cell death. Apoptosis.

[26] Hatsugai N, Kuroyanagi M, Yamada K, Meshi T, Tsuda S, Kondo M, Nishimura M, Hara-Nishimura I (2004). A plant vacuolar protease, VPE, mediates virus-induced hypersensitive cell death. Science.

[27] Nakaune S, Yamada K, Kondo M, Kato T, Tabata S, Nishimura M, Hara-Nishimura I (2005). A vacuolar processing enzyme, deltaVPE, is involved in seed coat formation at the early stage of seed development. Plant Cell.

[28] Kuroyanagi M, Yamada K, Hatsugai N, Kondo M, Nishimura M, Hara-Nishimura I (2005). Vacuolar processing enzyme is essential for mycotoxin-induced cell death in *Arabidopsis thaliana*. J Biol Chem.

[29] Jeong JS, Kim YS, Baek KH, Jung H, Ha SH, Do Choi Y, Kim M, Reuzeau C, Kim JK (2010). Root-specific expression of *OsNAC10* improves drought tolerance and grain yield in rice under field drought conditions. Plant Physiol.

[30] Du B, Zhang W, Liu B, Hu J, Wei Z, Shi Z, He R, Zhu L, Chen R, Han B (2009). Identification and characterization of *Bph14*, a gene conferring resistance to brown planthopper in rice. Proc Natl Acad Sci U S A.

[31] Deng H, Liu H, Li X, Xiao J, Wang S (2012). A CCCH-type zinc finger nucleic acid-binding protein quantitatively confers resistance against rice bacterial blight disease. Plant Physiol.

[32] Seo YS, Chern M, Bartley LE, Han M, Jung KH, Lee I, Walia H, Richter T, Xu X, Cao P (2011). Towards establishment of a rice stress response interactome. PLoS Genet.

[33] Wang Y, Zhu S, Liu S, Jiang L, Chen L, Ren Y, Han X, Liu F, Ji S, Liu X (2009). The vacuolar processing enzyme OsVPE1 is required for efficient glutelin processing in rice. Plant J.

[34] Beers EP, Jones AM, Dickerman AW (2004). The S8 serine, C1A cysteine and A1 aspartic protease families in *Arabidopsis*. Phytochemistry.

[35] Xia K, Liu T, Ouyang J, Wang R, Fan T, Zhang M (2011). Genome-wide identification, classification, and expression analysis of autophagy-associated gene homologues in rice (*Oryza sativa* L.). DNA Res.

[36] Hurst LD (2002). The Ka/Ks ratio: diagnosing the form of sequence evolution. Trends Genet.

[37] Petersen TN, Brunak S, von Heijne G, Nielsen H (2011). SignalP 4.0: discriminating signal peptides from transmembrane regions. Nat Methods.

[38] Bateman A, Bennett HP (2009). The granulin gene family: from cancer to dementia. BioEssays.

[39] Wang W, Zhao P, Zhou XM, Xiong HX, Sun MX (2015). Genome-wide identification and characterization of cystatin family genes in rice (*Oryza sativa* L.). Plant Cell Rep.

[40] Hara-Nishimura I, Kinoshita T, Hiraiwa N, Nishimura M (1998). Vacuolar processing enzymes in protein-storage vacuoles and lytic vacuoles. J Plant Physiol.

[41] Julian I, Gandullo J, Santos-Silva LK, Diaz I, Martinez M (2013). Phylogenetically distant barley legumains have a role in both seed and vegetative tissues. J Exp Bot.

[42] Okamoto T, Minamikawa T (1999). Molecular cloning and characterization of *Vigna mungo* processing enzyme 1 (VmPE-1), an asparaginyl endopeptidase possibly involved in post-translational processing of a vacuolar cysteine endopeptidase (SH-EP). Plant Mol Biol.

[43] Deng ZY, Gong CY, Wang T (2013). Use of proteomics to understand seed development in rice. Proteomics.

[44] Martinez M, Rubio-Somoza I, Carbonero P, Diaz I (2003). A cathepsin B-like cysteine protease gene from *Hordeum vulgare* (gene *CatB*) induced by GA in aleurone cells is under circadian control in leaves. J Exp Bot.

[45] Esteban-Garcia B, Garrido-Cardenas JA, Alonso DL, Garcia-Maroto F (2010). A distinct subfamily of papain-like cystein proteinases regulated by senescence and stresses in *Glycine max*. J Plant Physiol.

[46] Kinoshita T, Yamada K, Hiraiwa N, Kondo M, Nishimura M, Hara-Nishimura I (1999). Vacuolar processing enzyme is up-regulated in the lytic vacuoles of vegetative tissues during senescence and under various stressed conditions. Plant J.

[47] Yamada K, Nishimura M, Hara-Nishimura I (2004). The slow wound-response of *gammaVPE* is regulated by endogenous salicylic acid in *Arabidopsis*. Planta.

[48] Kamphuis IG, Drenth J, Baker EN (1985). Thiol proteases. Comparative studies based on the high-resolution structures of papain and actinidin, and on amino acid sequence information for cathepsins B and H, and stem bromelain. J Mol Biol.

[49] Karrer KM, Peiffer SL, Ditomas ME (1993). Two distinct gene subfamilies within the family of cysteine protease genes. Proc Natl Acad Sci U S A.

[50] Kramer L, Turk D, Turk B (2017). The future of cysteine cathepsins in disease management. Trends Pharmacol Sci.

[51] Coulombe R, Grochulski P, Sivaraman J, Menard R, Mort JS, Cygler M (1996). Structure of human procathepsin L reveals the molecular basis of inhibition by the prosegment. EMBO J.

[52] Zhao P, Zhou XM, Zhang LY, Wang W, Ma LG, Yang LB, Peng XB, Bozhkov PV, Sun MX (2013). A bipartite molecular module controls cell death activation in the basal cell lineage of plant embryos. PLoS Biol.

[53] Wan LL, Xia Q, Qiu X, Selvaraj G (2002). Early stages of seed development in *Brassica napus*: a seed coat-specific cysteine proteinase associated with programmed cell death of the inner integument. Plant J.

[54] Grudkowska M, Zagdanska B (2004). Multifunctional role of plant cysteine proteinases. Acta Biochim Pol.

[55] Zakharov A, Carchilan M, Stepurina T, Rotari V, Wilson K, Vaintraub I (2004). A comparative study of the role of the major proteinases of germinated common bean (*Phaseolus vulgaris* L.) and soybean (*Glycine max* (L.) Merrill) seeds in the degradation of their storage proteins. J Exp Bot.

[56] Ma L, Xin H, Qu L, Zhao J, Yang L, Zhao P, Sun M (2011). Transcription profile analysis reveals that zygotic division results in uneven distribution of specific transcripts in apical/basal cells of tobacco. PLoS One.

