# Supplementary material for: Papain-like and legumain-like proteases in rice: genome-wide identification, comprehensive gene feature characterization and expression analysis
Source: BMC Plant Biol. 2018 May 15;18:87. doi: 10.1186/s12870-018-1298-1 (PMC5952849; doi:10.1186/s12870-018-1298-1)
Supplement: Supplementary file 1 — Figure S1. Multiple protein sequences alignment of rice papain-like cysteine proteases. The inhibitor domain and peptidase C1A domain was shaded in red and black respectively. The granulin domain was marked with black boxes. The similar amino acid residues were marked in blue and the identical acid residues were boxed in purple. Red and black dots indicated the catalytic triad and the retention signal in ER respectively. The ‘NPIR’ was shaded in a black ellipse. (DOCX 868 kb) [file 12870_2018_1298_MOESM1_ESM.docx]

**
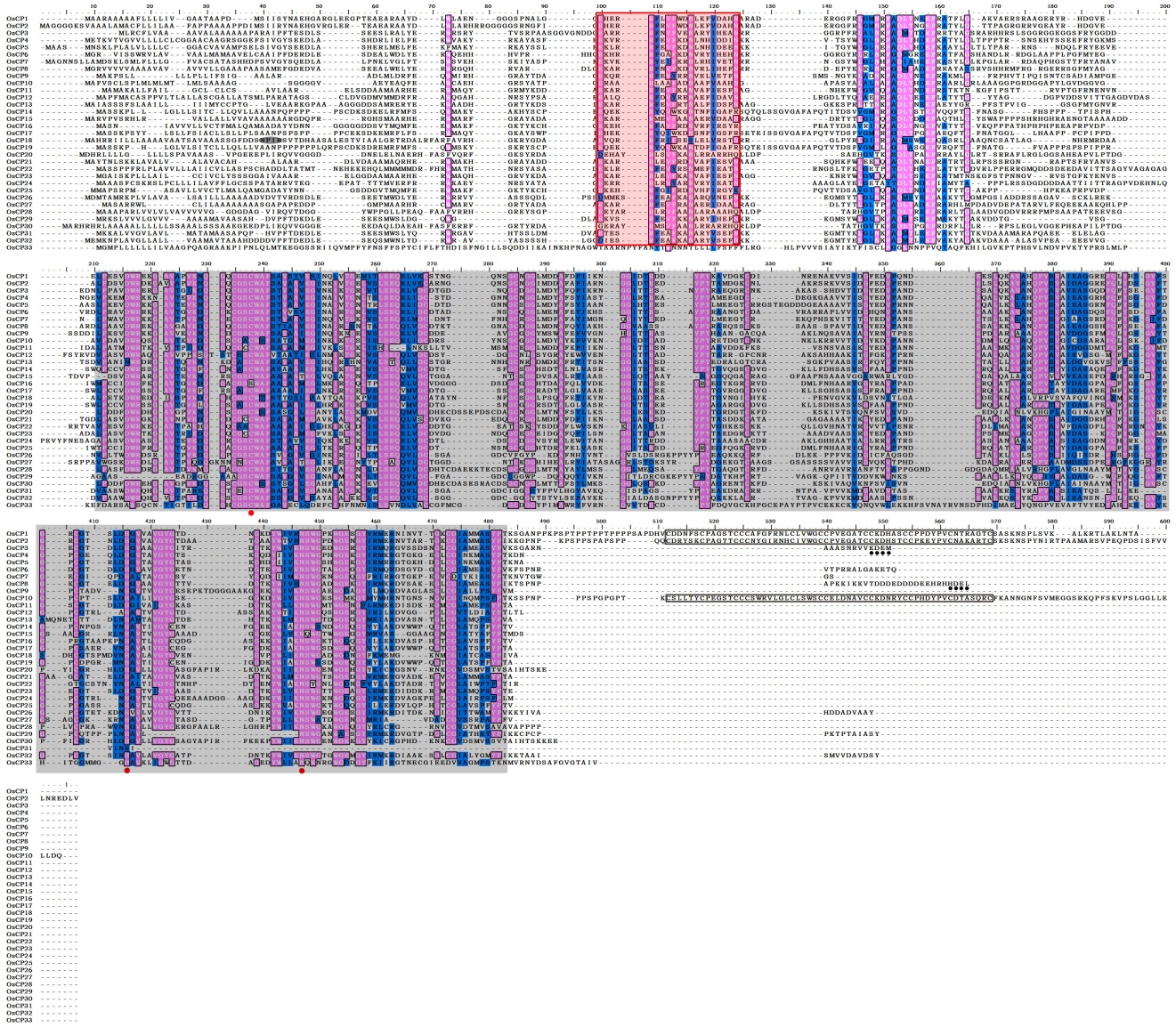
**

**Additional file 1: Figure S1** Multiple protein sequences alignment of rice papain-like cysteine proteases. The inhibitor domain and peptidase C1A domain was shaded in red and black respectively. The granulin domain was marked with black boxes. The similar amino acid residues were marked in blue and the identical acid residues were boxed in purple. Red and black dots indicated the catalytic triad and the retention signal in ER respectively. The ‘NPIR’ was shaded in a black ellipse
