# Supplementary material for: Papain-like and legumain-like proteases in rice: genome-wide identification, comprehensive gene feature characterization and expression analysis
Source: BMC Plant Biol. 2018 May 15;18:87. doi: 10.1186/s12870-018-1298-1 (PMC5952849; doi:10.1186/s12870-018-1298-1)
Supplement: Supplementary file 2 — Figure S2. Multiple protein sequences alignment of rice legumain-like cysteine proteases. The peptidase C13 domain was shaded. The similar amino acid residues were marked in blue and the identical acid residues were boxed in purple. Red dots indicate the catalytic triad. (DOCX 481 kb) [file 12870_2018_1298_MOESM2_ESM.docx]

**
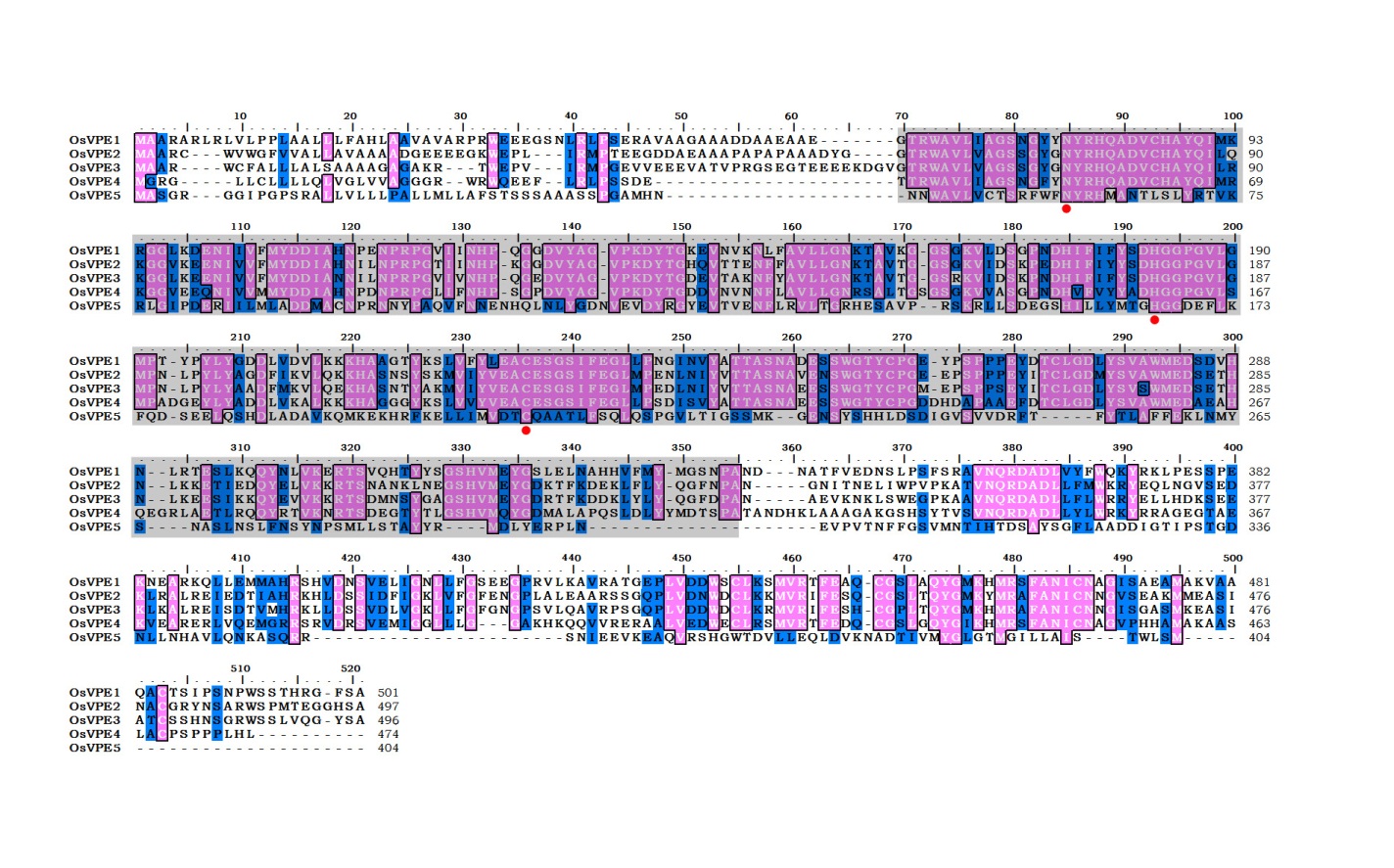
**

**Additional file 2: Figure S2** Multiple protein sequences alignment of rice legumain-like cysteine proteases. The peptidase C13 domain was shaded. The similar amino acid residues were marked in blue and the identical acid residues were boxed in purple. Red dots indicate the catalytic triad
