# Supplementary material for: Papain-like and legumain-like proteases in rice: genome-wide identification, comprehensive gene feature characterization and expression analysis
Source: BMC Plant Biol. 2018 May 15;18:87. doi: 10.1186/s12870-018-1298-1 (PMC5952849; doi:10.1186/s12870-018-1298-1)
Supplement: Supplementary file 3 — Figure S3. Expression heatmap of OsCPs in different tissues under normal conditions. (DOCX 2617 kb) [file 12870_2018_1298_MOESM3_ESM.docx]

**
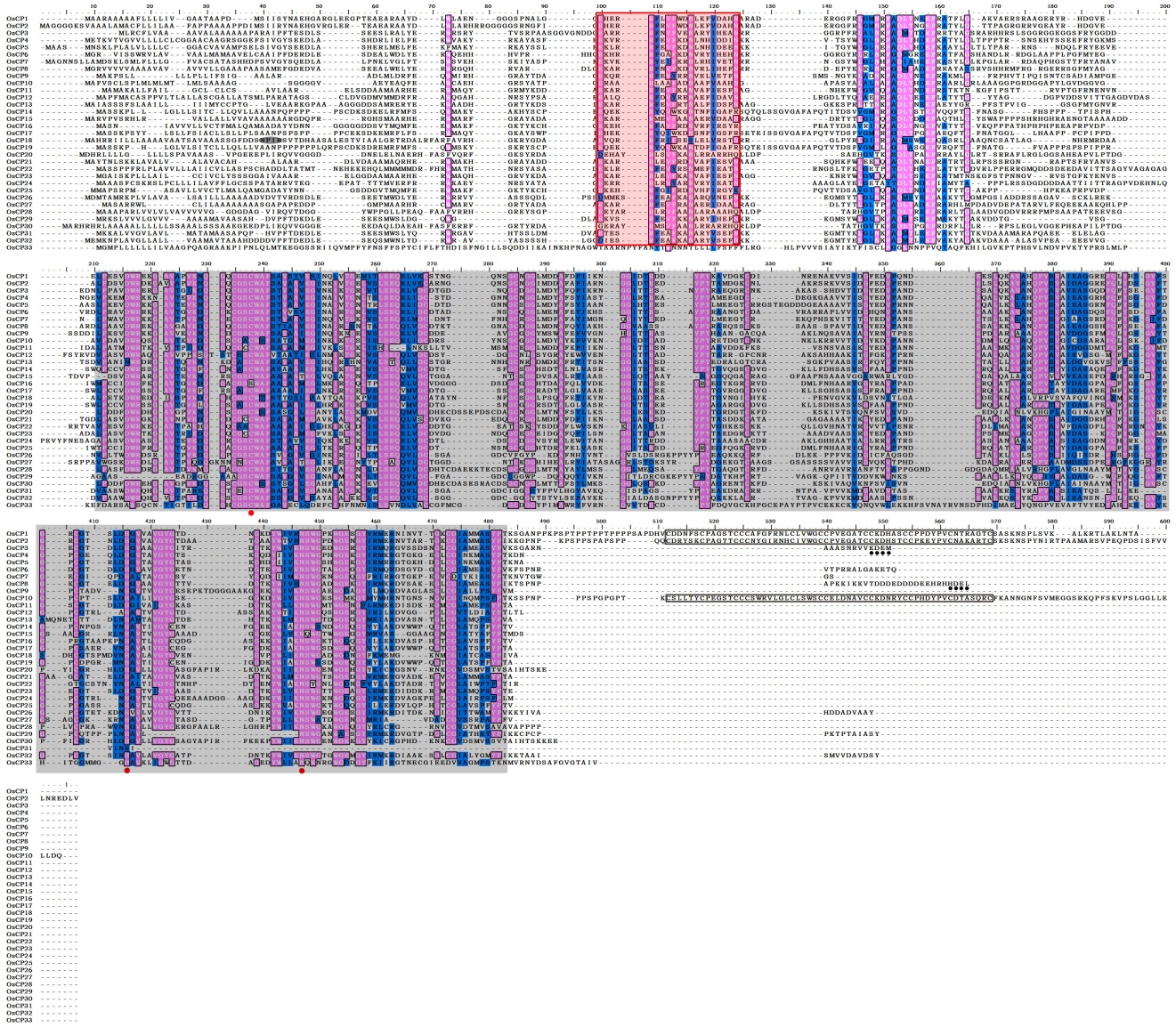
**

**Additional file 1: Figure S1** Multiple protein sequences alignment of rice papain-like cysteine proteases. The inhibitor domain and peptidase C1A domain was shaded in red and black respectively. The granulin domain was marked with black boxes. The similar amino acid residues were marked in blue and the identical acid residues were boxed in purple. Red and black dots indicated the catalytic triad and the retention signal in ER respectively. The ‘NPIR’ was shaded in a black ellipse

**
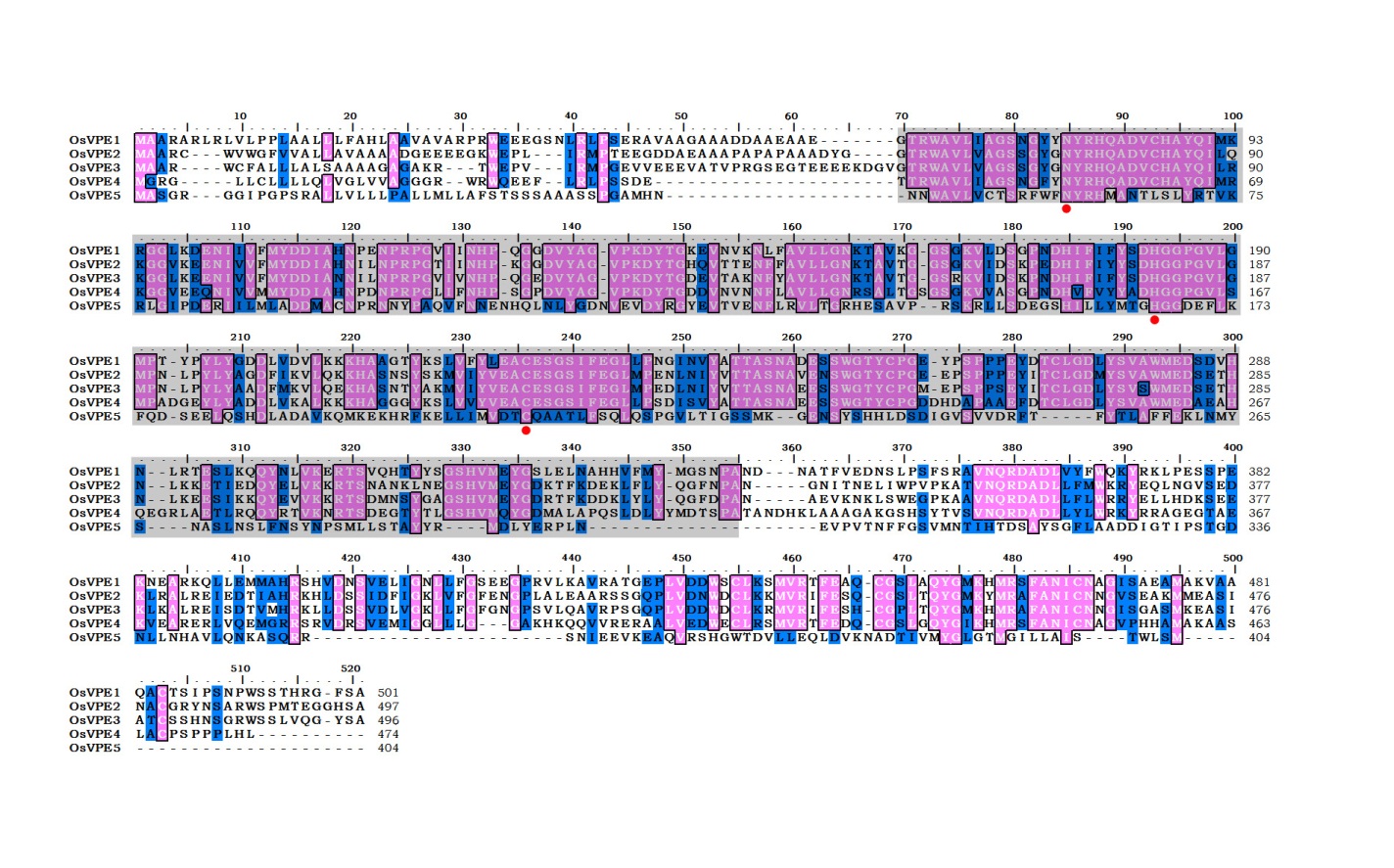
**

**Additional file 2: Figure S2** Multiple protein sequences alignment of rice legumain-like cysteine proteases. The peptidase C13 domain was shaded. The similar amino acid residues were marked in blue and the identical acid residues were boxed in purple. Red dots indicate the catalytic triad

**
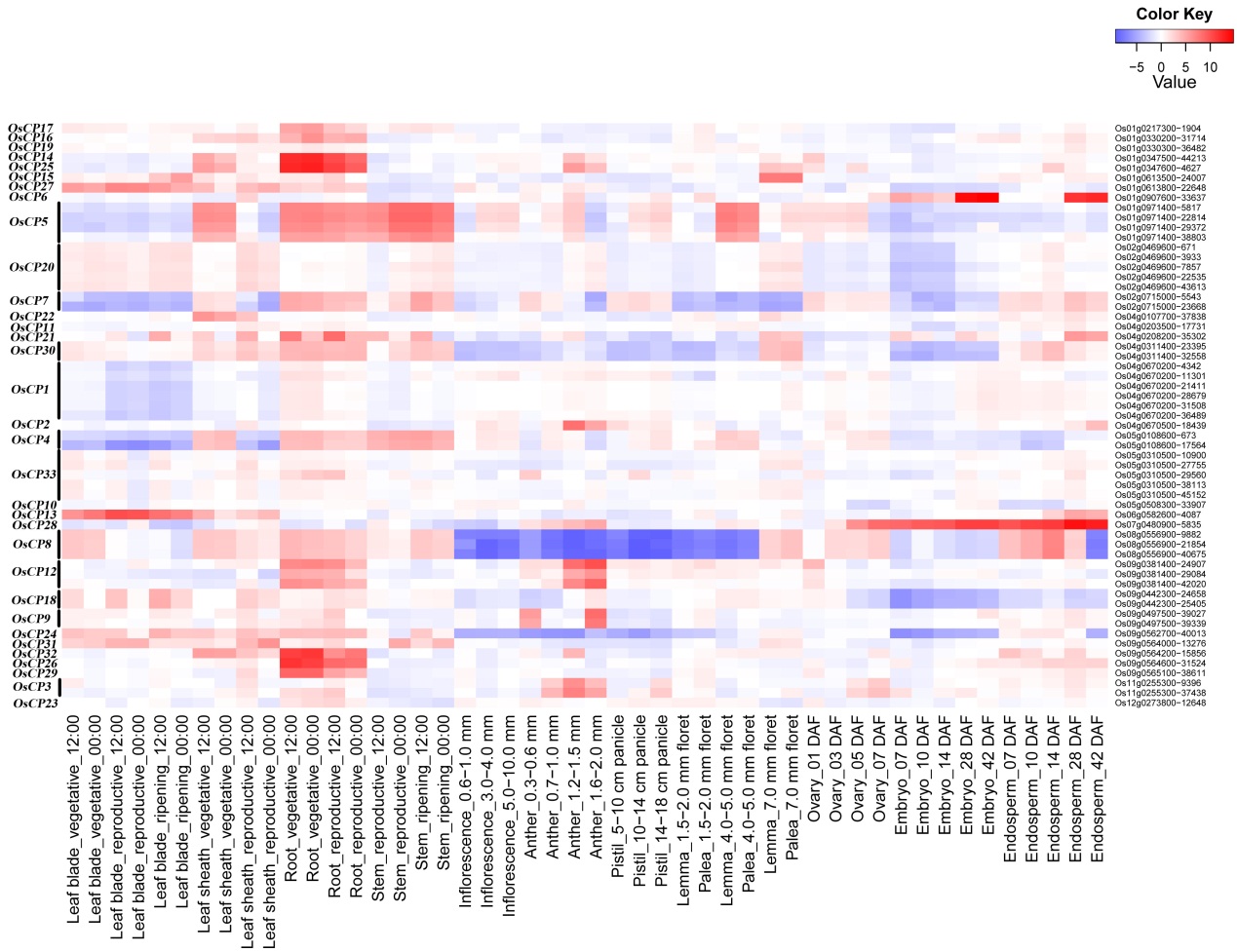
**

**Additional file 3: Figure S3** Expression heatmap of *OsCPs* in different tissues under normal conditions

**Table S4 Expression abundance of *OsCPs* and *OsVPEs* in various tissues under normal conditions**

| **Gene**  **name** | **14 days** | | **60 days** | | | | | | | | **60 days meristem**  **atic tissue** | **90 days**  **Immature**  **panicle** | **Mature**  **Pollen** | **Ovary and**  **Mature**  **stigma** | **3 days**  **Germin**  **ating**  **seed** | **35 days**  **Callus** |  |
| --- | --- | --- | --- | --- | --- | --- | --- | --- | --- | --- | --- | --- | --- | --- | --- | --- | --- |
|  | **Root** | **Leaf** | **Root** | **Root** | **Stem** | **Leaf** | | **Leaf** | **Leaf** | **Leaf** |  |  |  |  |  |  |  |
| *OsCP1* | 117 | 178 | 204 | 333 | 11 | 15 | |  |  |  | 55 | 279 | 126 | 2274 | 1093 | 401 |  |
| *OsCP3* |  |  |  |  |  |  | |  |  |  |  | 28 | 267 | 628 | 1558 |  |  |
| *OsCP4* |  |  | 4 |  | 48 |  | |  |  |  |  |  |  |  | 7 |  |  |
| *OsCP5* | 85 |  | 295 | 109 | 413 |  | |  |  |  | 526 | 1132 |  |  | 111 |  |  |
| *OsCP6* |  |  |  |  |  | 60 | |  |  |  |  |  |  |  | 569 | 5 |  |
| *OsCP7* |  |  |  |  |  |  | |  |  |  | 4 |  |  |  |  |  |  |
| *OsCP8* | 53 | 96 | 324 | 35 | 130 |  | |  |  |  | 212 |  |  | 102 | 7 |  |  |
| *OsCP9* | 200 |  |  |  |  |  | |  | 13 |  |  | 1 | 312 |  |  |  |  |
| *OsCP10* |  | 2 |  |  |  |  | |  |  | 20 | 5 | 6 | 7 |  | 7 | 18 |  |
| *OsCP12* | 42 |  |  |  |  |  | |  |  |  |  | 45 | 247 |  |  |  |  |
| *OsCP15* |  |  |  |  |  |  | |  |  |  |  |  |  | 51 |  |  |  |
| *OsCP16* |  |  |  |  |  |  | |  |  |  |  |  |  |  | 26 |  |  |
| *OsCP17* | 5 |  | 1 |  |  |  | |  |  |  |  |  |  |  | 5 |  |  |
| *OsCP18* | 298 | 156 | 52 | 68 | 25 | 2 | | 56 |  | 15 |  | 232 |  | 145 | 19 | 1 |  |
| *OsCP20* | 1454 | 2239 | 3255 | 1035 | 1004 | 3744 | | 808 | 1041 | 826 | 3337 | 2610 | 1047 | 1239 | 1251 | 726 |  |
| *OsCP21* |  |  | 7 |  |  |  | |  |  |  |  |  |  |  |  |  |  |
| *OsCP22* |  |  |  |  |  |  | |  |  |  |  |  |  |  | 23 |  |  |
| *OsCP25* | 190 |  | 45 | 153 |  |  | |  |  |  |  |  |  |  | 5 |  |  |
| *OsCP26* | 130 |  | 13 | 11 |  |  | |  |  |  |  |  |  |  |  |  |  |
| *OsCP27* |  | 111 | 5 |  |  |  | |  |  | 43 |  |  |  |  | 2 |  |  |
| *OsCP30* | 76 |  | 287 |  |  | 30 | |  | 8 |  |  |  |  |  | 23 |  |  |
| *OsCP31* |  | 7 |  |  | 1 | 4 | |  | 2 | 7 |  | 2 |  |  |  |  |  |
| *OsCP33* |  | 200 | 169 | 190 |  |  | |  | 79 | 122 | 145 | 35 | 224 | 242 | 793 | 124 |  |
| *OsVPE1* | 1105 | 3491 | 2902 | 1007 | 282 | 5111 | | 6587 | 3575 | 2830 | 519 | 192 | 156 | 88 | 502 | 197 |  |
| *OsVPE2* | 22 | 85 | 4 | 5 | 3 | |  | 113 |  |  | 33 |  |  | 115 | 93 | 90 | |
| *OsVPE3* | 88 | 4 | 118 | 202 | 2 | | 173 | 238 | 131 | 119 | 102 |  |  | 47 | 49 | 224 | |
| *OsVPE4* | 2 | 16 | 65 | 12 | 1 | |  |  |  |  | 2 |  |  |  | 32 |  | |
| *OsVPE5* |  | 66 |  |  |  | |  |  |  |  |  | 42 |  | 50 |  | 31 | |
| *Actin1* | 408 | 77 | 231 | 440 | 646 | | 45 | 92 | 223 | 174 | 205 | 666 | 2873 | 379 | 959 | 267 | |

**
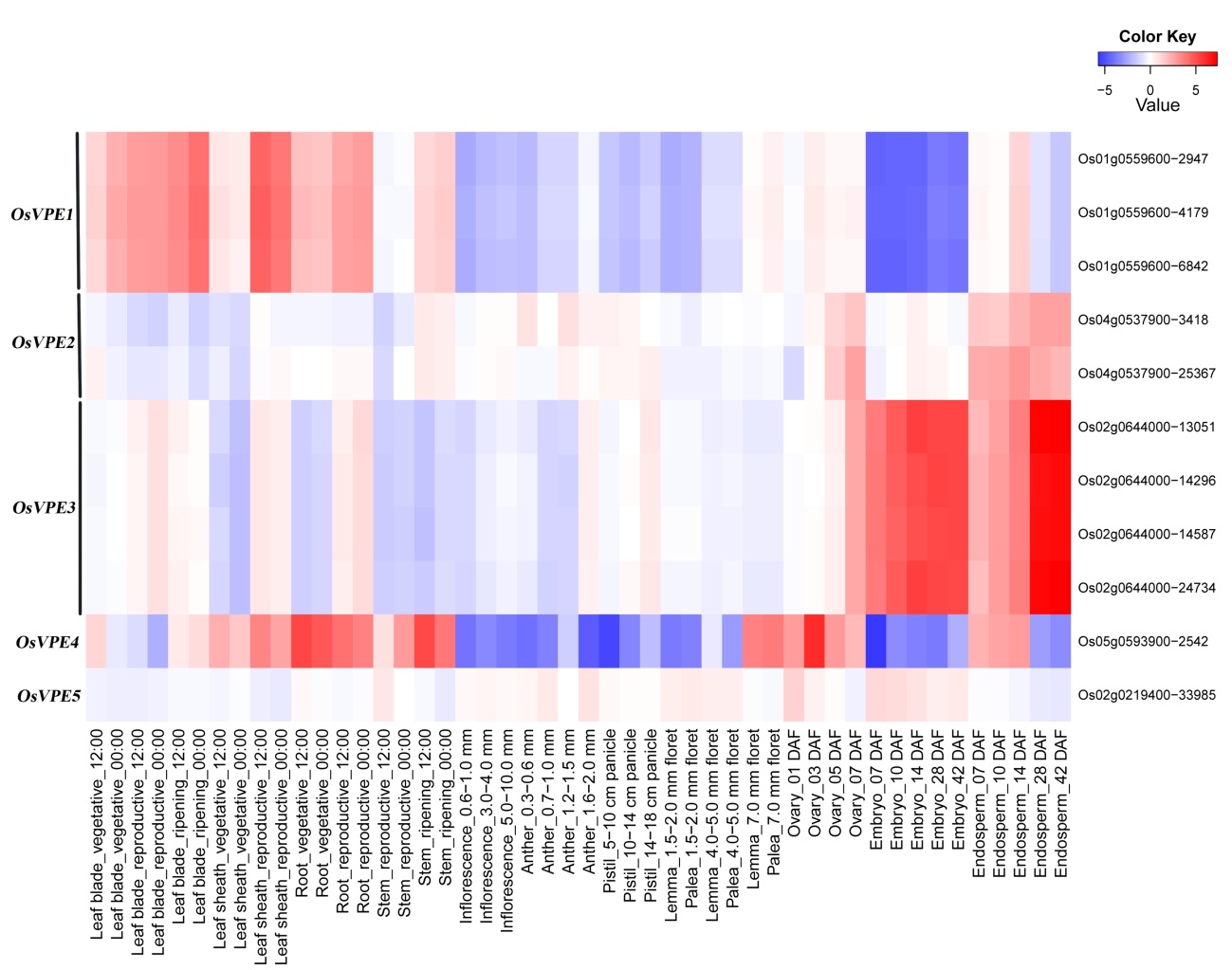
Additional file 5: Figure S5** Expression heatmap of *OsVPEs* in different tissues under normal conditions

**
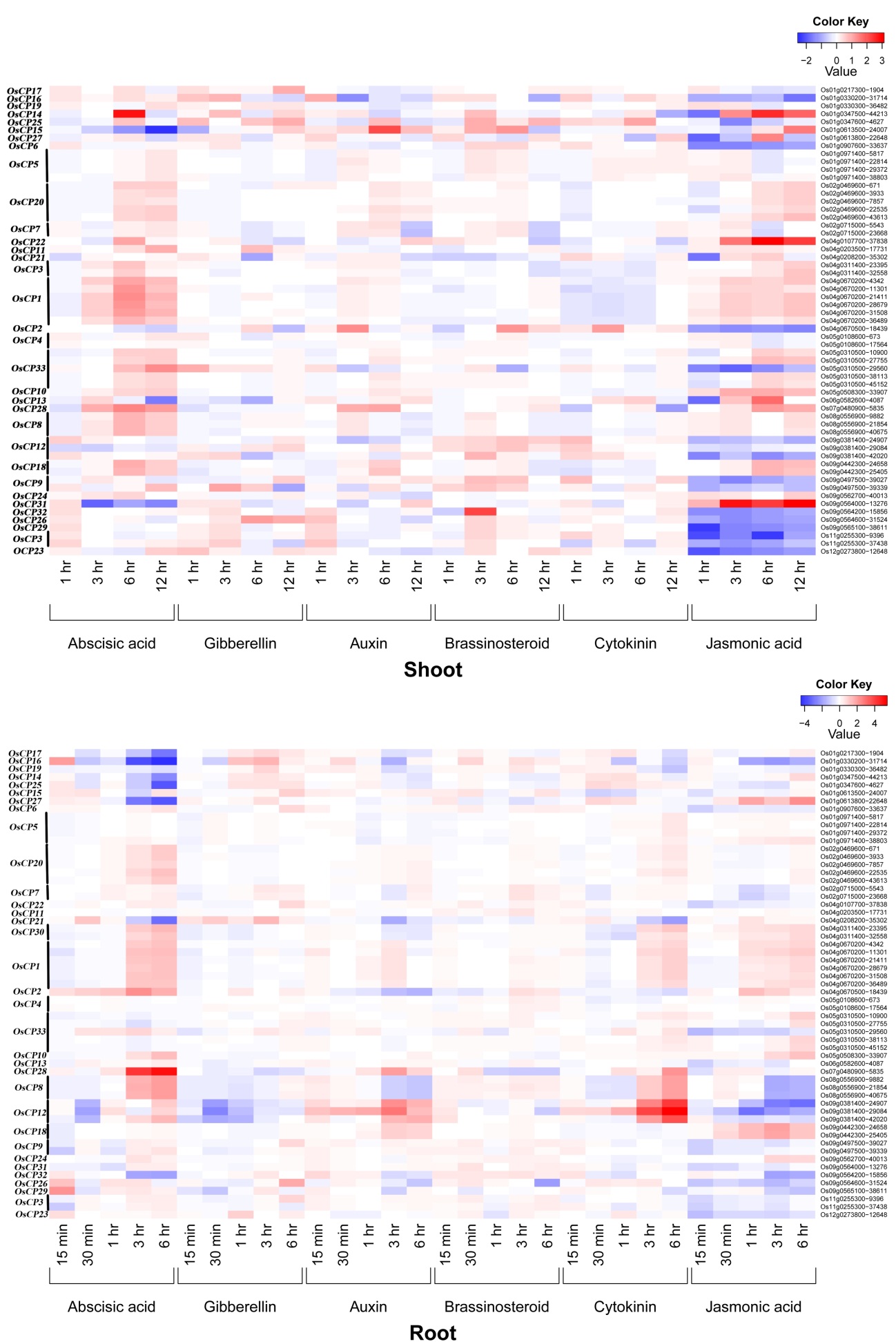
 Additional file 6: Figure S6** Expression profile of *OsCPs* in the shoots and roots under different plant hormones treatments

**Table S7 Expression change of *OsCPs* and *OsVPEs* under stress treatments**

| Gene  name | NACL | | DR | | COLD | |
| --- | --- | --- | --- | --- | --- | --- |
|  | Root | Leaf | Root | Leaf | Root | Leaf |
| *OsCP1* | 4.06 | 1.17 | 3.43 | 0.07 | 0.40 | 0.51 |
| *OsCP5* | 1.13 | - | 2.94 | - | 5.19 | - |
| *OsCP8* | 0.85 | 1.43 | 6.34 | 0.18 | 1.49 | 0.50 |
| *OsCP12* | 0.31 | - | - | -- | - | - |
| *OsCP18* | 0.18 | 2.49 | 1.15 | 0.56 | - | 0.57 |
| *OsCP20* | 1.33 | 1.46 | 2.44 | 1.57 | 0.46 | 0.17 |
| *OsCP25* | 2.85 | - | 0.14 | - | 0.41 | - |
| *OsCP26* | 1.00 | - | - | - | - | - |
| *OsCP27* | - | 0.25 | - | - | - | - |
| *OsCP30* | 2.34 | - | 2.55 | - | - | - |
| *OsCP31* | - | 4.57 | - | - | - | - |
| *OsCP33* | - | 1.27 | - | 0.51 | - | - |

| *OsVPE1* | 1.10 | 0.63 | 2.37 | 1.89 | 0.25 | 0.09 |
| --- | --- | --- | --- | --- | --- | --- |
| *OsVPE2* |  | 0.62 | 0.14 | 0.06 |  | 1.21 |
| *OsVPE3* | 1.06 | 36.50 | 1.51 | 3.75 | 0.17 | 0.50 |
| *OsVPE4* | 1.50 | 0.75 | 42.50 |  |  |  |
| *OsVPE5* |  |  |  | 0.12 |  |  |

The date was from MPSS: gene analysis (<http://mpss.udel.edu/rice/GeneQuery.php>). The experiment materials were 14-day-old seedlings. Salt treatment: 250 mM NACL for 24h; drought treatment: stressed in drought for 5d; cold treatment: 4℃ for 24h. Compared with normal condition, the expression fold change >2 or <0.5 were indicated in red or blue respectively

**Table S8 Overview of** **cis-elements in the promoters of *OsCPs* and *OsVPEs***

| Gene | ABRE  (ACGTGG/TC) | LTRE-core-motif  (G/ACCGAC) | DRE-core-motif  (A/GCCGAC) | T/G-box  (CACGTG/T) |
| --- | --- | --- | --- | --- |
| *OsCP1* | + | +++ | ++ | ++++ |
| *OsCP2* |  | + | + | + |
| *OsCP3* |  |  |  | + |
| *OsCP4* | + |  |  | ++ |
| *OsCP5* |  |  |  | ++ |
| *OsCP6* |  | + |  |  |
| *OsCP7* |  | + | + |  |
| *OsCP8* |  |  |  |  |
| *OsCP9* | + | + |  |  |
| *OsCP10* |  | +++ | + | ++ |
| *OsCP11* | + |  |  | + |
| *OsCP12* |  | + |  |  |
| *OsCP13* |  | ++++ | ++ |  |
| *OsCP14* |  | + | + | + |
| *OsCP15* |  |  |  |  |
| *OsCP16* |  |  |  | ++ |
| *OsCP17* | + | + | + |  |
| *OsCP18* |  | + |  | + |
| *OsCP19* |  | + | + | + |
| *OsCP20* |  |  |  | + |
| *OsCP21* |  | + |  | + |
| *OsCP22* |  |  |  | + |
| *OsCP23* |  |  |  |  |
| *OsCP24* |  | ++ |  |  |
| *OsCP25* |  |  |  |  |
| *OsCP26* |  |  |  |  |
| *OsCP27* |  |  |  | + |
| *OsCP28* | ++ |  |  | ++ |
| *OsCP29* | ++ |  |  | + |
| *OsCP30* |  | + |  | + |
| *OsCP31* |  |  |  |  |
| *OsCP32* |  |  |  | ++ |
| *OsCP33* | + | +++ | + |  |
| *OsVPE1* |  |  |  | + |
| *OsVPE2* | + | + | + | ++ |
| *OsVPE3* |  |  |  |  |
| *OsVPE4* |  |  |  |  |
| *OsVPE5* |  | ++++ |  |  |

ABRE: ABA-responsive element; LTRE: Low-temperature-responsive element; DRE: Dethydration-responsive-element; T/G-box: DNA-binding motif of MYC2 (the key transcriptional activator of jasmonate responses). The number of predicted cis-elements were presented by the number of “+”

**Table S9 Papain-like Cysteine Proteases in three plant species**

| Papain-like Cysteine Proteases | | | | | |
| --- | --- | --- | --- | --- | --- |
| *Arabidopsis thaliana* | | *Hordeum vulgare* | | *Zea mays* | |
| *AtCP1* | At1g47128 | *HvCP1* | BN000093 | *ZmCP1* | {162460343} |
| *AtCP2* | At5g43060 | *HvCP2* | AM941116 | *ZmCP2* | {162459488} |
| *AtCP3* | At4g36880 | *HvCP3* | AM941117 | *ZmCP3* | {162463334} |
| *AtCP4* | At3g19390 | *HvCP4* | AM941118 | *ZmCP4* | {238007404} |
| *AtCP5* | At3g19400 | *HvCP5* | AM941119 | *ZmCP5* | {162459393} |
| *AtCP6* | At3g43960 | *HvCP6* | AM941120 | *ZmCP6* | {194689248} |
| *AtCP7* | At4g11310 | *HvCP7* | AM941121 | *ZmCP7* | {226495425} |
| *AtCP8* | At4g11320 | *HvCP8* | AM941122 | *ZmCP8* | {195624522} |
| *AtCP9* | At4g23520 | *HvCP9* | U94591 | *ZmCP9* | {162463464} |
| *AtCP10* | At1g09850 | *HvCP10* | U19384 | *ZmCP10* | {226496089} |
| *AtCP11* | At3g48340 | *HvCP11* | U19359 | *ZmCP11* | {226501480} |
| *AtCP12* | At3g48350 | *HvCP12* | X05167 | *ZmCP12* | {238006338} |
| *AtCP13* | At5g50260 | *HvCP13* | AM941123 | *ZmCP13* | {226529105} |
| *AtCP14* | At1g20850 | *HvCP14* | AM941124 | *ZmCP14* | {226507950} |
| *AtCP15* | At4g35350 | *HvCP15* | AM941125 | *ZmCP15* | {195637152} |
| *AtCP16* | At5g45890 | *HvCP16* | AM941126 | *ZmCP16* | {226506492} |
| *AtCP17* | At1g06260 | *HvCP17* | Z97022 | *ZmCP17* | {226503129} |
| *AtCP18* | At2g34080 | *HvCP18* | AK251286 | *ZmCP18* | {226533314} |
| *AtCP19* | At1g29080 | *HvCP19* | AJ310426 | *ZmCP19* | {226505708} |
| *AtCP20* | At1g29090 | *HvCP20* | AM941127 | *ZmCP20* | {293334761} |
| *AtCP21* | At2g27420 | *HvCP21* | AM941128 | *ZmCP21* | {226509942} |
| *AtCP22* | At3g49340 | *HvCP22* | AM941129 | *ZmCP22* | {219884655} |
| *AtCP23* | At4g39090 | *HvCP23* | AM941130 | *ZmCP23* | {226503205} |
| *AtCP24* | At2g21430 | *HvCP24* | AM941131 | *ZmCP24* | {226531284} |
| *AtCP25* | At4g16190 | *HvCP25* | AM941132 | *ZmCP25* | {226499884} |
| *AtCP26* | At3g54940 | *HvCP26* | AM941133 | *ZmCP26* | {194701748} |
| *AtCP27* | At5g60360 | *HvCP27* | AK251383 | *ZmCP27* | {226507844 |
| *AtCP28* | At3g45310 | *HvCP28* | AM941134 | *ZmCP28* | {226504984} |
| *AtCP29* | At1g02305 | *HvCP29* | AK248562 | *ZmCP29* | {226508570} |
| *AtCP30* | At4g01610 | *HvCP30* | AK248416 | *ZmCP30* | {195628596} |
| *AtCP31* | At1g29110 | *HvCP31* | AK249908 | *ZmCP31* | {212275830} |
| *AtCP32* | At1g02300 | *HvCP32* | AK250687 | *ZmCP32* | {226502454} |
|  |  |  |  | *ZmCP33* | {194705198} |
|  |  |  |  | *ZmCP34* | {162459555} |
|  |  |  |  | *ZmCP35* | {1706261} |
|  |  |  |  | *ZmCP36* | (226497010} |

**Table S10 Legumain-like Cysteine Proteases in four plant species**

| VPEs（Legumain-like Cysteine Proteases） | | | | | | | |
| --- | --- | --- | --- | --- | --- | --- | --- |
| *Arabidopsis thaliana* | | *Hordeum vulgare* | | *Zea mays* | | *Glycine max* | |
| *AtVPE1* | At2g25940 | *HvVPE1* | AM941111 | *ZmVPE1* | AFW72544.1 | *GmVPE1* | XP_003525979.1 |
| *AtVPE2* | At1g62710 | *HvVPE2* | AM941112 | *ZmVPE2* | ACF79136.1 | *GmVPE2* | NP_001236564.1 |
| *AtVPE3* | At3g20210 | *HvVPE3* | AM941113 | *ZmVPE3* | ACG34144.1 | *GmVPE3* | XP_003550283.1 |
| *AtVPE4* | At4g32940 | *HvVPE4* | AM941114 | *ZmVPE4* | CAB64545.1 | *GmVPE4* | XP_006578073.1 |
|  |  | *HvVPE5* | AM941115 | *ZmVPE5* | NP_001105119.1 | *GmVPE5* | NP_001238297.1 |
|  |  |  |  | *ZmVPE6* | NP_001105613.1 | *GmVPE6* | NP_001236678.1 |
|  |  |  |  | *ZmVPE7* | CAC18100.1 |  |  |
|  |  |  |  | *ZmVPE8* | DAA36669.1 |  |  |
|  |  |  |  | *ZmVPE9* | NP_001241716.1 |  |  |


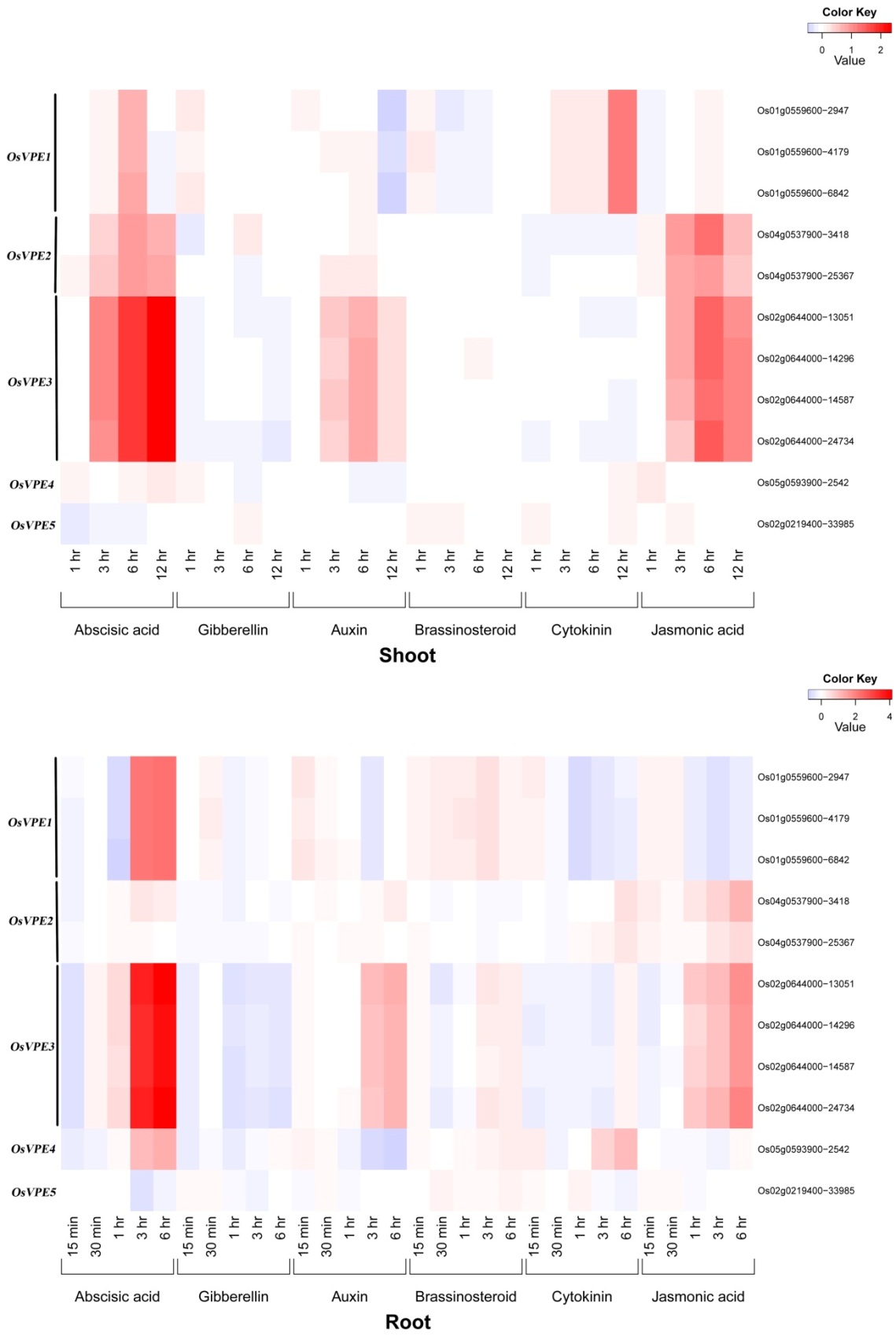


**Additional file 11: Figure S11** Expression profile of *OsVPEs* in the shoots and roots under different plant hormones treatments

**Table S12 primers used in this study**

| Name | Forward (5’-3’) | Reverse (5’-3’) |
| --- | --- | --- |
| *OsCP1* | GCTCAACCAATGGACAGAACA | CACCAGCCTCAATAGCAACAC |
| *OsCP2* | CCAAGAGCAAGAACAGCCCG | CTTCCCTATTCAAAACTACAAAAGAGAT |
| *OsCP3* | ACAGAAAGCAGTCGCAAACCA | TGAAGACACCCTCCGAATAGAA |
| *OsCP4* | CGCCTTCTCCTACATCGCCTC | GGACCATCAAACACACCCCCG |
| *OsCP5* | TACAGCGGGGGTGTGTTTGAT | GCGTTCTTGGTTGGGTAGGAG |
| *OsCP6* | ACATCCACGAGCACAACAAGC | ATGCCCTCCACGGACACCACC |
| *OsCP7* | AACTGGGGAGAGCAAGGGTAT | CGTTTTTGGTTGGGTAAGAAGC |
| *OsCP8* | CATCCGCACCAGCAACCTCA | GTTCGCCACCGCCTTCTTCA |
| *OsCP9* | AAACCATCCTTGCTCCTCCTT | GCATCTTGGCTCTGAACTCCTC |
| *OsCP10* | GGGTGAAAGTTGGGGTATGAAA | AGGGCAATAGGTAAGAAGGCTG |
| *OsCP11* | GTGGTTGTTGTTGGGCGTTTT | TCGTCTACTGCTGCGTATGGG |
| *OsCP12* | GCGACCTCACTTACCAGCTC | TGATCATGTTCAGGCTCTCG |
| *OsCP13* | CATAAACTGGAGGGATAGAGGCG | TTGCTGGTGATGTAGCGGAAC |
| *OsCP14* | TGGAGCAACGACTGGGGTGA | CGTGGGGTAGAATGGGGAGG |
| *OsCP15* | GTGGTCGTGGTCGTGGAGG | GGGTAGAAGGCGTAGGTGGC |
| *OsCP16* | AGTTCGTCGCCACCTACACC | ATCTTCATCAGCCCCTCCAT |
| *OsCP17* | TTCTCTTCTCCATCGCTTGCC | CCTCATCTCCTTGTCGGACTTCT |
| *OsCP18* | GCTCGTCCGCTCCACCAA | CCCATCCTCCCTCCAGTCTTT |
| *OsCP19* | TTGTCTTGTCCATCACTTGCCT | TACCGCTTCTCCTGCTCCTC |
| *OsCP20* | GGGCGAGAACTGGGGAGAGCA | GATGGCAGACACGGTGGAGAC |
| *OsCP21* | TTCGCCGACCTCACCAAC | CGAGAACGCCCAGCAACAC |
| *OsCP22* | TATGTGTCCTTTTGGCTTCTCCT | TGCTCTTTCTCGTGCTCGTTT |
| *OsCP23* | GCCAACGACGAGCCCAGC | CCCCACGAGTTCTTCACCAG |
| *OsCP24* | TACACCGACCTCACCAACGAC | TATCCCCTCCACCACCGCCAC |
| *OsCP25* | CAGGGTCTTGCTGGGCGT | CCGTTGCTGTTGGTGTCG |
| *OsCP26* | TGTCCGTTATCATCCAAATCAGC | GTCACGCCATACCCTACCACTA |
| *OsCP27* | GCGTCGTCCTCGTCCGTCG | GTGGTTCCGCTTCTTCCCG |
| *OsCP28* | GCCAACTTCCTCGCCACC | GCACTCCGTCTTCTTCTCCG |
| *OsCP29* | GTGGTGCTGGGTGTGGTGGTG | TGTCGGCGAACTTGTTGAGGC |
| *OsCP30* | AGAACTGGGGGGAGAAGGGGTA | TTTTTGGAGGTATGGATGGCA |
| *OsCP31* | ATCGGCGGCGGGTCGTATTTC | GCTGGAGGCTTTGGGTGGCTT |
| *OsCP32* | CAGGGTGGGGTGAGATGGGTT | GCAGCGGTTTTCTTGATGGGG |
| *OsCP33* | ATGTCCCTGTCAAAACTTATCCA | CCATCACAACCATCGCCGC |
| *OsVPE1* | GCTACTACAACTACCGCCACCA | CCGAGCAGAACAGCAAACAA |
| *OsVPE2* | AGTTACTGGAGGGAGTGGGAAG | AAAGATACGGCAGGTTAGGCATA |
| *OsVPE3* | CATAGATAGCAAACCAAATGACCAC | AAATACTGCCACTTTCACACGC |
| *OsVPE4* | GTGGACAGAAGCGTGGAGATGA | TAAGTGTAACGGAGGGGGGGAT |
| *OsVPE5* | AGTAAAGCAAATGAAGGAGAAGCA | GGTAAACCTATCCACAACAGAAACA |
| *ACTIN* | CTTCATAGGAATGGAAGCTGCGGGTA | CGACCACCTTGATCTTCATGCTGCTA |
| *UBC* | CCGTTTGTAGAGCCATAATTGCA | AGGTTGCCTGAGTCACAGTTAAGTG |
