# Supplementary material for: Papain-like and legumain-like proteases in rice: genome-wide identification, comprehensive gene feature characterization and expression analysis
Source: BMC Plant Biol. 2018 May 15;18:87. doi: 10.1186/s12870-018-1298-1 (PMC5952849; doi:10.1186/s12870-018-1298-1)
Supplement: Supplementary file 4 — Table S4. Expression abundance of OsCPs and OsVPEs in various tissues under normal conditions. (DOCX 20 kb) [file 12870_2018_1298_MOESM4_ESM.docx]

**Table S4 Expression abundance of *OsCPs* and *OsVPEs* in various tissues under normal conditions**

| **Gene**  **name** | **14 days** | | **60 days** | | | | | | | | **60 days meristem**  **atic tissue** | **90 days**  **Immature**  **panicle** | **Mature**  **Pollen** | **Ovary and**  **Mature**  **stigma** | **3 days**  **Germin**  **ating**  **seed** | **35 days**  **Callus** |  |
| --- | --- | --- | --- | --- | --- | --- | --- | --- | --- | --- | --- | --- | --- | --- | --- | --- | --- |
|  | **Root** | **Leaf** | **Root** | **Root** | **Stem** | **Leaf** | | **Leaf** | **Leaf** | **Leaf** |  |  |  |  |  |  |  |
| *OsCP1* | 117 | 178 | 204 | 333 | 11 | 15 | |  |  |  | 55 | 279 | 126 | 2274 | 1093 | 401 |  |
| *OsCP3* |  |  |  |  |  |  | |  |  |  |  | 28 | 267 | 628 | 1558 |  |  |
| *OsCP4* |  |  | 4 |  | 48 |  | |  |  |  |  |  |  |  | 7 |  |  |
| *OsCP5* | 85 |  | 295 | 109 | 413 |  | |  |  |  | 526 | 1132 |  |  | 111 |  |  |
| *OsCP6* |  |  |  |  |  | 60 | |  |  |  |  |  |  |  | 569 | 5 |  |
| *OsCP7* |  |  |  |  |  |  | |  |  |  | 4 |  |  |  |  |  |  |
| *OsCP8* | 53 | 96 | 324 | 35 | 130 |  | |  |  |  | 212 |  |  | 102 | 7 |  |  |
| *OsCP9* | 200 |  |  |  |  |  | |  | 13 |  |  | 1 | 312 |  |  |  |  |
| *OsCP10* |  | 2 |  |  |  |  | |  |  | 20 | 5 | 6 | 7 |  | 7 | 18 |  |
| *OsCP12* | 42 |  |  |  |  |  | |  |  |  |  | 45 | 247 |  |  |  |  |
| *OsCP15* |  |  |  |  |  |  | |  |  |  |  |  |  | 51 |  |  |  |
| *OsCP16* |  |  |  |  |  |  | |  |  |  |  |  |  |  | 26 |  |  |
| *OsCP17* | 5 |  | 1 |  |  |  | |  |  |  |  |  |  |  | 5 |  |  |
| *OsCP18* | 298 | 156 | 52 | 68 | 25 | 2 | | 56 |  | 15 |  | 232 |  | 145 | 19 | 1 |  |
| *OsCP20* | 1454 | 2239 | 3255 | 1035 | 1004 | 3744 | | 808 | 1041 | 826 | 3337 | 2610 | 1047 | 1239 | 1251 | 726 |  |
| *OsCP21* |  |  | 7 |  |  |  | |  |  |  |  |  |  |  |  |  |  |
| *OsCP22* |  |  |  |  |  |  | |  |  |  |  |  |  |  | 23 |  |  |
| *OsCP25* | 190 |  | 45 | 153 |  |  | |  |  |  |  |  |  |  | 5 |  |  |
| *OsCP26* | 130 |  | 13 | 11 |  |  | |  |  |  |  |  |  |  |  |  |  |
| *OsCP27* |  | 111 | 5 |  |  |  | |  |  | 43 |  |  |  |  | 2 |  |  |
| *OsCP30* | 76 |  | 287 |  |  | 30 | |  | 8 |  |  |  |  |  | 23 |  |  |
| *OsCP31* |  | 7 |  |  | 1 | 4 | |  | 2 | 7 |  | 2 |  |  |  |  |  |
| *OsCP33* |  | 200 | 169 | 190 |  |  | |  | 79 | 122 | 145 | 35 | 224 | 242 | 793 | 124 |  |
| *OsVPE1* | 1105 | 3491 | 2902 | 1007 | 282 | 5111 | | 6587 | 3575 | 2830 | 519 | 192 | 156 | 88 | 502 | 197 |  |
| *OsVPE2* | 22 | 85 | 4 | 5 | 3 | |  | 113 |  |  | 33 |  |  | 115 | 93 | 90 | |
| *OsVPE3* | 88 | 4 | 118 | 202 | 2 | | 173 | 238 | 131 | 119 | 102 |  |  | 47 | 49 | 224 | |
| *OsVPE4* | 2 | 16 | 65 | 12 | 1 | |  |  |  |  | 2 |  |  |  | 32 |  | |
| *OsVPE5* |  | 66 |  |  |  | |  |  |  |  |  | 42 |  | 50 |  | 31 | |
| *Actin1* | 408 | 77 | 231 | 440 | 646 | | 45 | 92 | 223 | 174 | 205 | 666 | 2873 | 379 | 959 | 267 | |
