# Supplementary material for: Papain-like and legumain-like proteases in rice: genome-wide identification, comprehensive gene feature characterization and expression analysis
Source: BMC Plant Biol. 2018 May 15;18:87. doi: 10.1186/s12870-018-1298-1 (PMC5952849; doi:10.1186/s12870-018-1298-1)
Supplement: Supplementary file 5 — Figure S5. Expression heatmap of OsVPEs in different tissues under normal conditions. (DOCX 263 kb) [file 12870_2018_1298_MOESM5_ESM.docx]

**
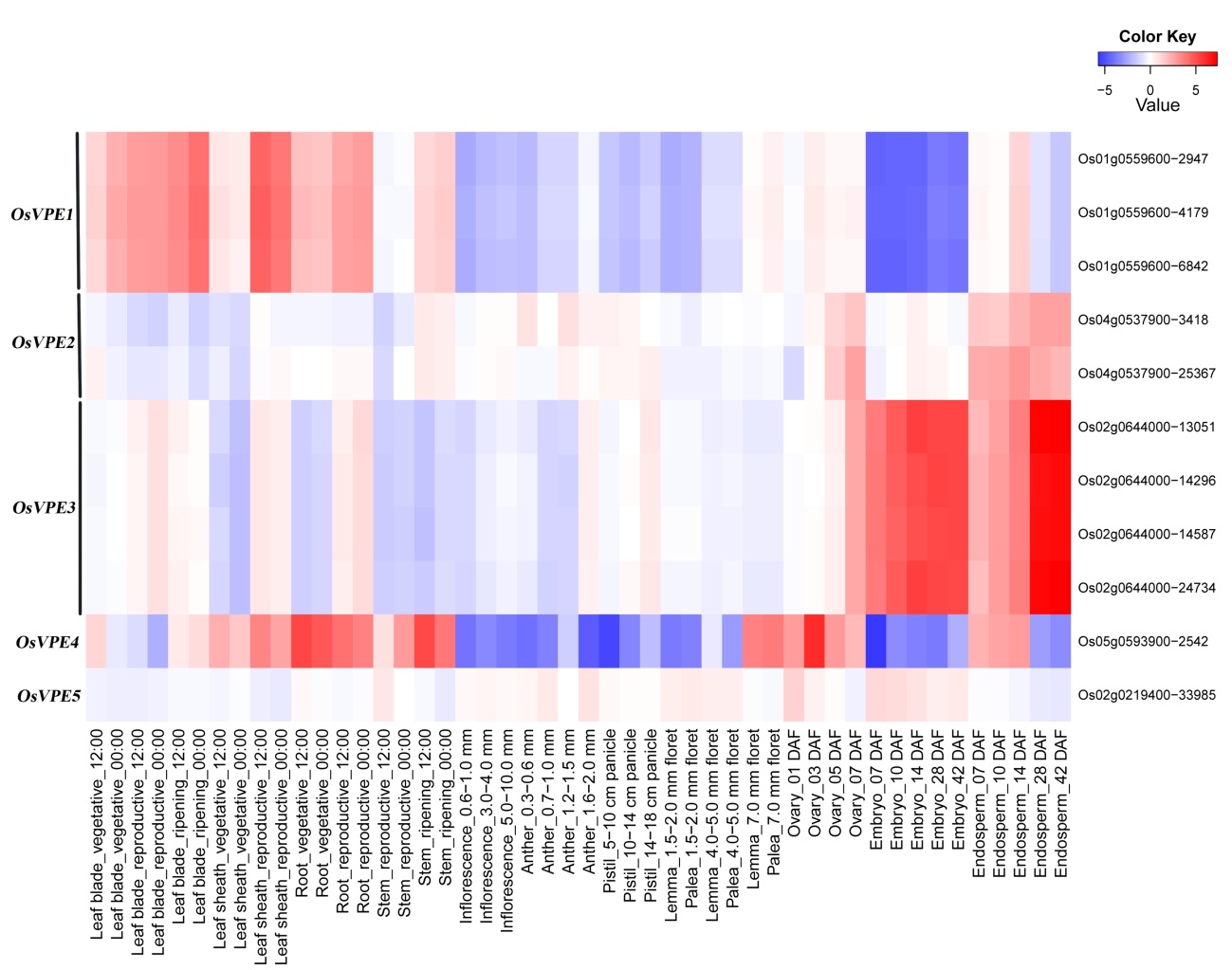
Additional file 5: Figure S5** Expression heatmap of *OsVPEs* in different tissues under normal conditions
