# Supplementary material for: Papain-like and legumain-like proteases in rice: genome-wide identification, comprehensive gene feature characterization and expression analysis
Source: BMC Plant Biol. 2018 May 15;18:87. doi: 10.1186/s12870-018-1298-1 (PMC5952849; doi:10.1186/s12870-018-1298-1)
Supplement: Supplementary file 6 — Figure S6. Expression profile of OsCPs in the shoots and roots under different plant hormones treatments. (DOCX 482 kb) [file 12870_2018_1298_MOESM6_ESM.docx]

**
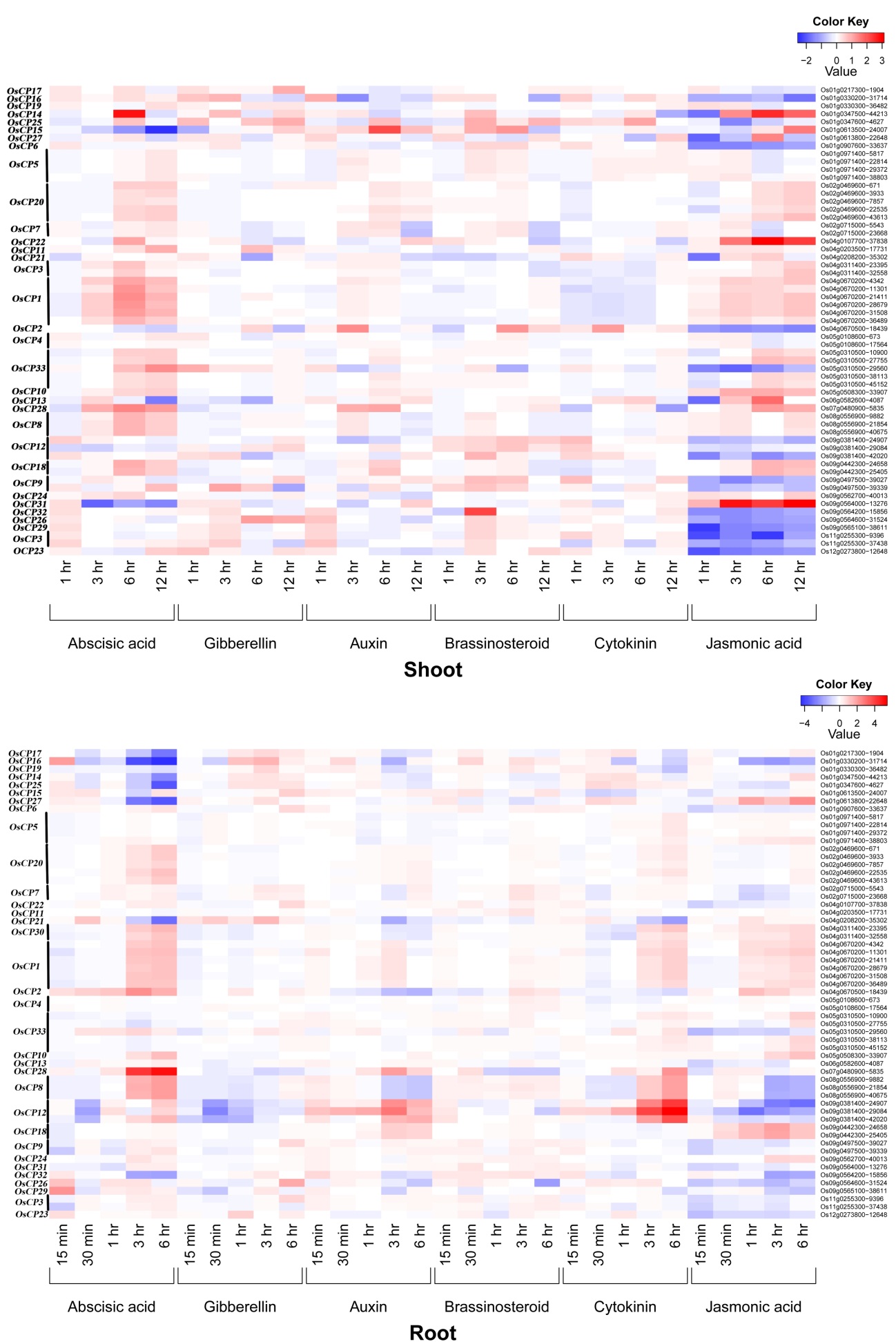
 Additional file 6: Figure S6** Expression profile of *OsCPs* in the shoots and roots under different plant hormones treatments
