# Supplementary material for: Papain-like and legumain-like proteases in rice: genome-wide identification, comprehensive gene feature characterization and expression analysis
Source: BMC Plant Biol. 2018 May 15;18:87. doi: 10.1186/s12870-018-1298-1 (PMC5952849; doi:10.1186/s12870-018-1298-1)
Supplement: Supplementary file 7 — Table S7. Expression change of OsCPs and OsVPEs under stress treatments. The date was from MPSS: gene analysis (http://mpss.udel.edu/rice/GeneQuery.php). The experiment materials were 14-day-old seedlings. Salt treatment: 250 mM NACL for 24 h; drought treatment: stressed in drought for 5d; cold treatment: 4 °C for 24 h. Compared with normal condition, the expression fold change > 2 or < 0.5 were indicated in red or blue respectively. (DOCX 16 kb) [file 12870_2018_1298_MOESM7_ESM.docx]

**Table S7 Expression change of *OsCPs* and *OsVPEs* under stress treatments**

| Gene  name | NACL | | DR | | COLD | |
| --- | --- | --- | --- | --- | --- | --- |
|  | Root | Leaf | Root | Leaf | Root | Leaf |
| *OsCP1* | 4.06 | 1.17 | 3.43 | 0.07 | 0.40 | 0.51 |
| *OsCP5* | 1.13 | - | 2.94 | - | 5.19 | - |
| *OsCP8* | 0.85 | 1.43 | 6.34 | 0.18 | 1.49 | 0.50 |
| *OsCP12* | 0.31 | - | - | -- | - | - |
| *OsCP18* | 0.18 | 2.49 | 1.15 | 0.56 | - | 0.57 |
| *OsCP20* | 1.33 | 1.46 | 2.44 | 1.57 | 0.46 | 0.17 |
| *OsCP25* | 2.85 | - | 0.14 | - | 0.41 | - |
| *OsCP26* | 1.00 | - | - | - | - | - |
| *OsCP27* | - | 0.25 | - | - | - | - |
| *OsCP30* | 2.34 | - | 2.55 | - | - | - |
| *OsCP31* | - | 4.57 | - | - | - | - |
| *OsCP33* | - | 1.27 | - | 0.51 | - | - |

| *OsVPE1* | 1.10 | 0.63 | 2.37 | 1.89 | 0.25 | 0.09 |
| --- | --- | --- | --- | --- | --- | --- |
| *OsVPE2* |  | 0.62 | 0.14 | 0.06 |  | 1.21 |
| *OsVPE3* | 1.06 | 36.50 | 1.51 | 3.75 | 0.17 | 0.50 |
| *OsVPE4* | 1.50 | 0.75 | 42.50 |  |  |  |
| *OsVPE5* |  |  |  | 0.12 |  |  |

The date was from MPSS: gene analysis (<http://mpss.udel.edu/rice/GeneQuery.php>). The experiment materials were 14-day-old seedlings. Salt treatment: 250 mM NACL for 24h; drought treatment: stressed in drought for 5d; cold treatment: 4℃ for 24h. Compared with normal condition, the expression fold change >2 or <0.5 were indicated in red or blue respectively
