# Supplementary material for: Papain-like and legumain-like proteases in rice: genome-wide identification, comprehensive gene feature characterization and expression analysis
Source: BMC Plant Biol. 2018 May 15;18:87. doi: 10.1186/s12870-018-1298-1 (PMC5952849; doi:10.1186/s12870-018-1298-1)
Supplement: Supplementary file 8 — Table S8. Overview of cis-elements in the promoters of OsCPs and OsVPEs. ABRE: ABA-responsive element; LTRE: Low-temperature-responsive element; DRE: Dethydration-responsive-element; T/G-box: DNA-binding motif of MYC2 (the key transcriptional activator of jasmonate responses). The number of predicted cis-elements were presented by the number of “+”. (DOCX 17 kb) [file 12870_2018_1298_MOESM8_ESM.docx]

**Table S8 Overview of** **cis-elements in the promoters of *OsCPs* and *OsVPEs***

| Gene | ABRE  (ACGTGG/TC) | LTRE-core-motif  (G/ACCGAC) | DRE-core-motif  (A/GCCGAC) | T/G-box  (CACGTG/T) |
| --- | --- | --- | --- | --- |
| *OsCP1* | + | +++ | ++ | ++++ |
| *OsCP2* |  | + | + | + |
| *OsCP3* |  |  |  | + |
| *OsCP4* | + |  |  | ++ |
| *OsCP5* |  |  |  | ++ |
| *OsCP6* |  | + |  |  |
| *OsCP7* |  | + | + |  |
| *OsCP8* |  |  |  |  |
| *OsCP9* | + | + |  |  |
| *OsCP10* |  | +++ | + | ++ |
| *OsCP11* | + |  |  | + |
| *OsCP12* |  | + |  |  |
| *OsCP13* |  | ++++ | ++ |  |
| *OsCP14* |  | + | + | + |
| *OsCP15* |  |  |  |  |
| *OsCP16* |  |  |  | ++ |
| *OsCP17* | + | + | + |  |
| *OsCP18* |  | + |  | + |
| *OsCP19* |  | + | + | + |
| *OsCP20* |  |  |  | + |
| *OsCP21* |  | + |  | + |
| *OsCP22* |  |  |  | + |
| *OsCP23* |  |  |  |  |
| *OsCP24* |  | ++ |  |  |
| *OsCP25* |  |  |  |  |
| *OsCP26* |  |  |  |  |
| *OsCP27* |  |  |  | + |
| *OsCP28* | ++ |  |  | ++ |
| *OsCP29* | ++ |  |  | + |
| *OsCP30* |  | + |  | + |
| *OsCP31* |  |  |  |  |
| *OsCP32* |  |  |  | ++ |
| *OsCP33* | + | +++ | + |  |
| *OsVPE1* |  |  |  | + |
| *OsVPE2* | + | + | + | ++ |
| *OsVPE3* |  |  |  |  |
| *OsVPE4* |  |  |  |  |
| *OsVPE5* |  | ++++ |  |  |

ABRE: ABA-responsive element; LTRE: Low-temperature-responsive element; DRE: Dethydration-responsive-element; T/G-box: DNA-binding motif of MYC2 (the key transcriptional activator of jasmonate responses). The number of predicted cis-elements were presented by the number of “+”
