# Supplementary material for: Papain-like and legumain-like proteases in rice: genome-wide identification, comprehensive gene feature characterization and expression analysis
Source: BMC Plant Biol. 2018 May 15;18:87. doi: 10.1186/s12870-018-1298-1 (PMC5952849; doi:10.1186/s12870-018-1298-1)
Supplement: Supplementary file 9 — Table S9. Papain-like Cysteine Proteases in three plant species. (DOCX 18 kb) [file 12870_2018_1298_MOESM9_ESM.docx]

**Table S9 Papain-like Cysteine Proteases in three plant species**

| Papain-like Cysteine Proteases | | | | | |
| --- | --- | --- | --- | --- | --- |
| *Arabidopsis thaliana* | | *Hordeum vulgare* | | *Zea mays* | |
| *AtCP1* | At1g47128 | *HvCP1* | BN000093 | *ZmCP1* | {162460343} |
| *AtCP2* | At5g43060 | *HvCP2* | AM941116 | *ZmCP2* | {162459488} |
| *AtCP3* | At4g36880 | *HvCP3* | AM941117 | *ZmCP3* | {162463334} |
| *AtCP4* | At3g19390 | *HvCP4* | AM941118 | *ZmCP4* | {238007404} |
| *AtCP5* | At3g19400 | *HvCP5* | AM941119 | *ZmCP5* | {162459393} |
| *AtCP6* | At3g43960 | *HvCP6* | AM941120 | *ZmCP6* | {194689248} |
| *AtCP7* | At4g11310 | *HvCP7* | AM941121 | *ZmCP7* | {226495425} |
| *AtCP8* | At4g11320 | *HvCP8* | AM941122 | *ZmCP8* | {195624522} |
| *AtCP9* | At4g23520 | *HvCP9* | U94591 | *ZmCP9* | {162463464} |
| *AtCP10* | At1g09850 | *HvCP10* | U19384 | *ZmCP10* | {226496089} |
| *AtCP11* | At3g48340 | *HvCP11* | U19359 | *ZmCP11* | {226501480} |
| *AtCP12* | At3g48350 | *HvCP12* | X05167 | *ZmCP12* | {238006338} |
| *AtCP13* | At5g50260 | *HvCP13* | AM941123 | *ZmCP13* | {226529105} |
| *AtCP14* | At1g20850 | *HvCP14* | AM941124 | *ZmCP14* | {226507950} |
| *AtCP15* | At4g35350 | *HvCP15* | AM941125 | *ZmCP15* | {195637152} |
| *AtCP16* | At5g45890 | *HvCP16* | AM941126 | *ZmCP16* | {226506492} |
| *AtCP17* | At1g06260 | *HvCP17* | Z97022 | *ZmCP17* | {226503129} |
| *AtCP18* | At2g34080 | *HvCP18* | AK251286 | *ZmCP18* | {226533314} |
| *AtCP19* | At1g29080 | *HvCP19* | AJ310426 | *ZmCP19* | {226505708} |
| *AtCP20* | At1g29090 | *HvCP20* | AM941127 | *ZmCP20* | {293334761} |
| *AtCP21* | At2g27420 | *HvCP21* | AM941128 | *ZmCP21* | {226509942} |
| *AtCP22* | At3g49340 | *HvCP22* | AM941129 | *ZmCP22* | {219884655} |
| *AtCP23* | At4g39090 | *HvCP23* | AM941130 | *ZmCP23* | {226503205} |
| *AtCP24* | At2g21430 | *HvCP24* | AM941131 | *ZmCP24* | {226531284} |
| *AtCP25* | At4g16190 | *HvCP25* | AM941132 | *ZmCP25* | {226499884} |
| *AtCP26* | At3g54940 | *HvCP26* | AM941133 | *ZmCP26* | {194701748} |
| *AtCP27* | At5g60360 | *HvCP27* | AK251383 | *ZmCP27* | {226507844 |
| *AtCP28* | At3g45310 | *HvCP28* | AM941134 | *ZmCP28* | {226504984} |
| *AtCP29* | At1g02305 | *HvCP29* | AK248562 | *ZmCP29* | {226508570} |
| *AtCP30* | At4g01610 | *HvCP30* | AK248416 | *ZmCP30* | {195628596} |
| *AtCP31* | At1g29110 | *HvCP31* | AK249908 | *ZmCP31* | {212275830} |
| *AtCP32* | At1g02300 | *HvCP32* | AK250687 | *ZmCP32* | {226502454} |
|  |  |  |  | *ZmCP33* | {194705198} |
|  |  |  |  | *ZmCP34* | {162459555} |
|  |  |  |  | *ZmCP35* | {1706261} |
|  |  |  |  | *ZmCP36* | (226497010} |
