# Supplementary material for: Papain-like and legumain-like proteases in rice: genome-wide identification, comprehensive gene feature characterization and expression analysis
Source: BMC Plant Biol. 2018 May 15;18:87. doi: 10.1186/s12870-018-1298-1 (PMC5952849; doi:10.1186/s12870-018-1298-1)
Supplement: Supplementary file 10 — Table S10. Legumain-like Cysteine Proteases in four plant species. (DOCX 16 kb) [file 12870_2018_1298_MOESM10_ESM.docx]

**Table S10 Legumain-like Cysteine Proteases in four plant species**

| VPEs（Legumain-like Cysteine Proteases） | | | | | | | |
| --- | --- | --- | --- | --- | --- | --- | --- |
| *Arabidopsis thaliana* | | *Hordeum vulgare* | | *Zea mays* | | *Glycine max* | |
| *AtVPE1* | At2g25940 | *HvVPE1* | AM941111 | *ZmVPE1* | AFW72544.1 | *GmVPE1* | XP_003525979.1 |
| *AtVPE2* | At1g62710 | *HvVPE2* | AM941112 | *ZmVPE2* | ACF79136.1 | *GmVPE2* | NP_001236564.1 |
| *AtVPE3* | At3g20210 | *HvVPE3* | AM941113 | *ZmVPE3* | ACG34144.1 | *GmVPE3* | XP_003550283.1 |
| *AtVPE4* | At4g32940 | *HvVPE4* | AM941114 | *ZmVPE4* | CAB64545.1 | *GmVPE4* | XP_006578073.1 |
|  |  | *HvVPE5* | AM941115 | *ZmVPE5* | NP_001105119.1 | *GmVPE5* | NP_001238297.1 |
|  |  |  |  | *ZmVPE6* | NP_001105613.1 | *GmVPE6* | NP_001236678.1 |
|  |  |  |  | *ZmVPE7* | CAC18100.1 |  |  |
|  |  |  |  | *ZmVPE8* | DAA36669.1 |  |  |
|  |  |  |  | *ZmVPE9* | NP_001241716.1 |  |  |
