# Supplementary material for: Papain-like and legumain-like proteases in rice: genome-wide identification, comprehensive gene feature characterization and expression analysis
Source: BMC Plant Biol. 2018 May 15;18:87. doi: 10.1186/s12870-018-1298-1 (PMC5952849; doi:10.1186/s12870-018-1298-1)
Supplement: Supplementary file 11 — Figure S11. Expression profile of OsVPEs in the shoots and roots under different plant hormones treatments. (DOCX 214 kb) [file 12870_2018_1298_MOESM11_ESM.docx]

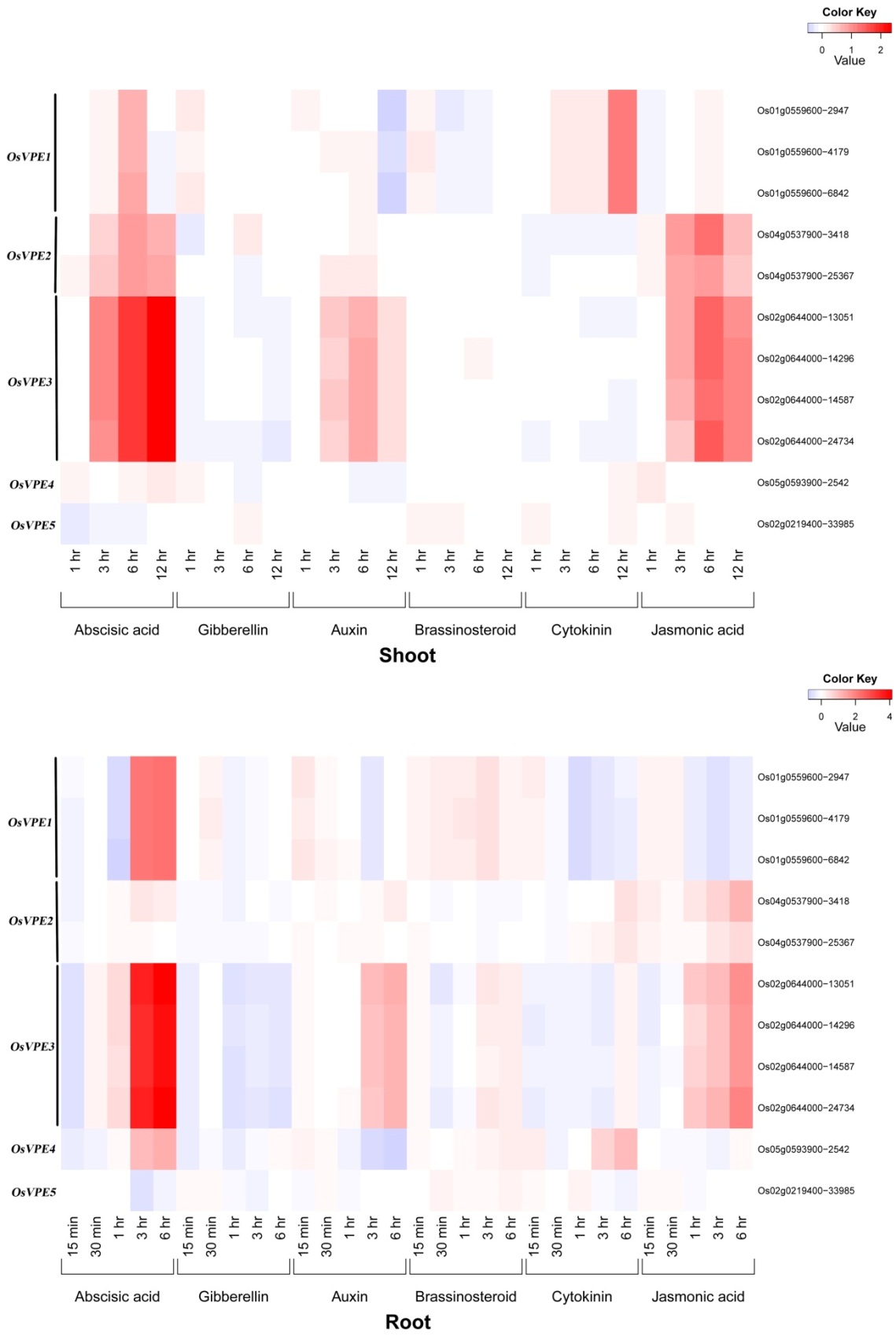


**Additional file 11: Figure S11** Expression profile of *OsVPEs* in the shoots and roots under different plant hormones treatments
